# Engineering CLL-1 CAR-NK cells via mRNA–LNP for potent antitumor activity and reversal of HLA-E–mediated resistance in acute myeloid leukemia

**DOI:** 10.1186/s13046-026-03689-4

**Published:** 2026-03-17

**Authors:** Jie Shen, Peng Jin, Yebing Liang, Jinyang Zhu, Rufang Xiang, Zeyi Li, Hongming Zhu, Xiaoyang Li, Yunxiang Zhang, Wei Wang, Zhen Jin, Junmin Li

**Affiliations:** 1 Shanghai Institute of Hematology, State Key Laboratory of Medical Genomics, National Research Center for Translational Medicine at Shanghai, Ruijin Hospital Affiliated to Shanghai Jiao Tong University School of Medicine, Shanghai, China; 2 Shanghai Xinpu BioTechnology Company Limited, Shanghai, China; 3 Department of General Practice, Ruijin Hospital, Shanghai Jiao Tong University School of Medicine, Shanghai, China; 4 Department of Pharmacy and Pharmaceutical Science, St. Jude Children’s Research Hospital, Memphis, TN USA

**Keywords:** MRNA, Lipid nanoparticles, Acute myeloid leukemia, Chimeric antigen receptor, Natural killer cells, Multidrug resistance

## Abstract

**Background:**

Acute myeloid leukemia (AML) remains a highly lethal malignancy, with relapse primarily driven by resistance to chemotherapy or targeted therapies. Existing chimeric antigen receptor T cell (CAR-T) strategies are limited by toxicity and complex manufacturing, underscoring the need for transient, controllable, and safe CAR-engineering platforms that can selectively target multidrug-resistant (MDR) AML cells.

**Methods:**

We developed a lipid nanoparticle (LNP)–based mRNA delivery platform for scalable generation of C-type lectin-like molecule-1 (CLL-1) CAR-NK cells. NK phenotype, cytotoxicity, cytokine secretion, and safety were evaluated ex vivo against AML cell lines and patient-derived blasts, with in vivo efficacy tested in xenograft NSG mouse models. Mechanisms of adaptive resistance were investigated through transcriptomic profiling, modulation of the NKG2A/HLA-E axis, and functional interrogation of the JAK2–STAT1 signaling pathway.

**Results:**

Drug-response profiling across AML cohorts identified a multidrug-resistant subgroup marked by a distinct transcriptomic program in which CLL-1 was the only validated CAR target upregulated. mRNA–LNP transfection enabled efficient generation of primary CLL-1 CAR-NK cells with preserved phenotype and potent, antigen-specific cytotoxicity against AML cells, while sparing normal hematopoietic progenitors. In vivo, repeated CAR-NK infusions markedly suppressed leukemia progression and prolonged survival. Transcriptomic analyses of tumor cells surviving CAR-NK exposure revealed inflammatory activation with progressive HLA-E upregulation, which impaired CAR-NK function. NKG2A blockade restored cytotoxicity ex vivo and enhanced leukemia clearance and survival in vivo. Mechanistically, prolonged CAR-NK engagement or IFN-γ stimulation activated a JAK2–STAT1 axis that drove sustained HLA-E induction. JAK2 knockdown reduced HLA-E expression and sensitized AML cells to CAR-NK–mediated killing, whereas pharmacologic JAK2 inhibition also decreased HLA-E expression but concurrently impaired NK-cell activation, thereby limiting the overall therapeutic benefit.

**Conclusions:**

Transient, non-integrating mRNA–LNP–transfected CLL-1 CAR-NK cells provide a safe and effective strategy for MDR AML. Repeated dosing enables robust antitumor activity, while adaptive resistance via NKG2A/HLA-E axis can be mitigated through checkpoint blockade. The JAK2–STAT1 pathway represents a potential upstream modulator, providing opportunities for rational combinatorial approaches to optimize CAR-NK therapy.

**Supplementary Information:**

The online version contains supplementary material available at 10.1186/s13046-026-03689-4.

## Introduction

Acute Myeloid Leukemia (AML) is an aggressive hematologic malignancy with a median age at 68 years [1]. Nearly half of newly diagnosed patients are unable to tolerate intensive chemotherapy, resulting in a poor overall prognosis, largely due to the inability to proceed to allogeneic hematopoietic stem cell transplantation [2–4]. Although the frontline combination therapy of venetoclax and azacitidine (Ven/Aza) has improved complete remission rates to nearly 70% in recent years, the durability of responses remains limited [5, 6]. Salvage regimens of chemotherapy combining targeted agents have demonstrated minimal clinical benefit, underscoring a substantial unmet therapeutic need.

Cellular immunotherapy, particularly chimeric antigen receptor-T cell (CAR-T) therapy, has demonstrated substantial clinical efficacy in relapsed or refractory B-cell lymphomas and multiple myeloma, in part by harnessing mechanisms that circumvent conventional pathways of multidrug resistance [7–11]. C-type lectin-like molecule-1 (CLL-1) is a lineage-specific antigen expressed in approximately 77–92% of AML patients [12, 13], with stable expression across disease status and lack expression on normal hematopoietic stem cells (HSCs) [12, 14, 15]. Among reported clinical trials, CAR-T therapy targeting CLL-1 has yielded high remission rates, highlighting its promise as a therapeutic strategy for AML [6, 16]. In this study, we further identified that CLL-1 expression was increased in multidrug-resistant (MDR) AML populations, suggesting that CLL-1-targeted cellular immunotherapy might overcome the resistance to first-line therapies and expand salvage treatment options of AML patients.

Despite these advances, conventional CAR-T cell therapy is associated with cytokine release syndrome (CRS), immune effector cell-associated neurotoxicity syndrome (ICANS), and the potential for secondary T-cell malignancies, all of which remain major clinical concerns in patients with hematologic malignancies [17, 18]. Moreover, the high cost manufacturing further constrains its accessibility [19]. In addition, cytokines within the bone marrow microenvironment have been implicated in mediating resistance to CAR-T therapy in AML [20]. Compared with healthy donors, NK and T cells derived from AML patients often exhibit impaired functionality [20, 21]. As a result, “off-the-shelf” CAR natural killer (CAR-NK) cell therapy has gained increasing attention [19]. Specifically, CAR-NK cells selectively recognize and eliminate AML cells based on tumor-associated antigens, while exhibiting lower toxicity than CAR-T cells and minimal risk of inducing CRS or ICANS [22–24]. Although the persistence of CAR-NK cells in vivo was not comparable to CAR-T cells, this limitation can be mitigated through repeated infusion, with no increase in overall adverse events or higher toxicity. [22] Off-the-shelf CAR-NK cells also circumvent HLA-matching constraints and effectively modulated the immunosuppressive AML microenvironment, thereby enhancing anti-leukemic efficacy without the need for patient-specific manufacturing. [25–27].

However, significant technical hurdles remain in CAR-NK cell manufacturing. Viral transduction efficiency is low and variable (19–73%), while nonviral approaches can compromise NK viability [28–33]. In recent years, messenger RNA (mRNA)-based CAR delivery has shown substantial promise in CAR-T cell applications. Transient mRNA-based CAR expression addresses concerns regarding long-term off-target toxicity associated with conventional CAR therapies. It has been reported that anti-CD8 antibody-modified lipid nanoparticle (LNP)-like carriers enabled transient CAR mRNA expression in T cells, and tumor regression has been observed with repeated infusions [34]. Another study showed that LNP-mediated CAR mRNA delivery has reduced myocardial fibrosis. [35] These findings underscore the translational potential of LNP-mediated mRNA delivery as a clinically scalable and safe gene transfer platform.

Autologous NK cells from patients are often limited in number and exhibit functional impairment or exhaustion under disease conditions [36, 37]. Compared with peripheral blood from healthy donors, umbilical cord blood (CB) contains a higher proportion of NK cells, which are more developmentally immature, possess greater ex vivo expansion potential, and show relatively lower immunogenicity [38, 39]. Moreover, this source avoids the complex differentiation procedures and potential safety concerns in iPSC-derived NK products [40–42]. Therefore, cord blood–derived NK cells may represent a more suitable source for ex vivo LNP engineering and off-the-shelf therapeutic strategies due to their stable availability, expansion capacity, and scalability.

Here, we employed an LNP-based platform to transfect CLL-1 CAR mRNA into CB–derived primary NK cells, achieving high transfection efficiency with preserved viability and function. The resulting CLL-1 CAR-NK cells demonstrated potent cytotoxicity ex vivo and in vivo. Notably, sustained CAR-NK activity can induce human leukocyte antigen-E (HLA-E)–mediated adaptive resistance, and combination with the natural killer group 2 member A (NKG2A)-blocking antibody monalizumab effectively restored cytotoxicity. Given that IFN-γ–JAK2–STAT1 signaling contributes to HLA-E upregulation, our approach provides a framework not only for targeting AML through CAR-NK therapy but also for integrating strategies that modulate immune-evasion pathways, highlighting the potential for combinatorial interventions to further enhance therapeutic efficacy.

## Materials and methods

### Human sample collection and cell lines

CB-derived NK cells were obtained from Qilu Stem Cell Engineering Co., LTD. Bone marrow mononuclear cells (BMMCs) were collected from 11 AML patients, and peripheral blood mononuclear cells (PBMCs) were collected from 2 healthy donors at Ruijin Hospital affiliated to Shanghai Jiao Tong University School of Medicine. The collection of the specimens was approved by the Ruijin Hospital Ethics Committee, Shanghai Jiao Tong University School of Medicine (2024-LLSD-464), and the written informed consent for specimen collection and research was obtained following the Declaration of Helsinki. THP-1, HL-60, 293 T and Nalm-6 cells were kind gift from Dr. Rufang Xiang, and OCI-AML2 was provided by Procell Life Science & Technology Co., Ltd. They were cultured in RPMI 1640 medium, supplemented with 10% FBS and 1% Penicillin/Streptomycin.

### Define the MDR and multidrug-sensitive AML groups

To specifically evaluate ex vivo responses to first-line drugs in AML, we integrated publicly available drug sensitivity datasets from three sources: the Functional Precision Medicine Tumor Board (FPMTB) cohort (Zenodo: https://zenodo.org/records/7274740), the BeatAML cohort (https://biodev.github.io/BeatAML2/), and the study by Qin et al [43]. Drug response was quantified using selective drug sensitivity scores (sDSS) in the FPMTB cohort, where higher scores indicate greater sensitivity, and by area under the curve (AUC) in BeatAML and log_10_ (IC50) in Qin et al., where higher values reflect increased resistance.

Ex vivo drug-sensitivity profiles were clustered using the Ward.D2 method, as implemented in the pheatmap R package. This agglomerative clustering approach minimizes within-cluster variance, enabling clear separation of patient groups based on drug response patterns. Differential expression analysis between MDR and multidrug-sensitive groups was performed using the DESeq2 package. Differentially expressed genes (DEGs) were defined by a false discovery rate (FDR) < 0.05 and an absolute log_2_ fold change > 0.8.

### Antibodies and flow cytometric analysis

To evaluate NK purity and CAR expression efficiency, CAR-NK and untreated NK (NT-NK) cells were stained with the following antibodies: anti-CD56-APC, anti-CD3-FITC, anti-CD56-PE/Cyanine7, anti-CD3-Brilliant Violet 785 (both Biolegend), anti-F(ab’)_2_ fragment-Alexa Fluor 647 (Jackson Immunoresearch). For phenotyping, the following antibodies were used: anti-CD16-FITC, anti-CD57-PE/Cyanine7, anti-NKG2C-PE, anti-NKp46-PE/Cyanine7, anti-NKp30-PE, anti-NKp44-PE, anti-FasL-PE, anti-NKG2A-PE, anti-DNAM-1-PE, anti-NKG2D-PE/Cyanine7, anti-TIGIT-PE/Cyanine7, anti-LAG3-PE, anti-HLA-E-APC, anti-CD107a-PE/Dazzle 594, anti-CLL-1-PE, anti-CLL-1-APC, anti-CD13-APC, anti-CD14-FITC, anti-CD15-PE (all Biolegend). Catalog numbers are provided in the Supplementary Information. Data were analyzed using FlowJo (FlowJo 13.07.20).

### mRNA–LNP generation and transfection of CLL-1 CAR-NK cells

mRNA was synthesized in vitro using the Capped RNA Synthesis Kit (with tailing) (Hongene Biotech, ON-269) to generate capped transcripts. For template preparation, the CAR-NK coding sequence was cloned into a DNA plasmid backbone containing a T7 AG promoter upstream of the open reading frame, flanked by optimized 5′ and 3′ untranslated regions (UTRs) to enhance stability and translation efficiency, followed by a 100-nucleotide poly(A) tail downstream of the stop codon.

For in vitro transcription (IVT), the reaction mixture contained the DNA template, 5 × Reaction Buffer, 2 × ARCA/NTP mix, 10 × poly(A) polymerase Reaction Buffer. Reactions were incubated at 37 °C for 2 h to allow efficient transcription and capping. Following transcription, residual DNA templates were digested with DNase I for 30 min at 37 °C. The IVT reaction was then followed by Poly(A) tailing procedure by adding Poly(A) polymerase to the solution and incubated for 30 min at 37 °C. Transcript integrity was confirmed by agarose gel electrophoresis against an RNA molecular weight ladder, and mRNA concentration was quantified spectrophotometrically using a NanoDrop spectrophotometer (Thermo Fisher Scientific, Waltham, MA, USA).

For encapsulation, purified mRNA was diluted in 100 mM citrate buffer (pH 4.0) and mixed with a lipid formulation containing ionizable lipid (50 mol%), DSPC (10 mol%), cholesterol (38.5 mol%), and DMG-PEG2000 (1.5 mol%), all dissolved in ethanol. Formulation was carried out on the NanoAssemblr™ Ignite™ nanoparticle formulation systems (Vancouver, British Columbia, Canada) at an aqueous:organic phase flow ratio of 4:1, which promotes spontaneous self-assembly of uniform LNPs. The resulting mRNA–LNP complexes were subsequently purified and concentrated using Amicon® Ultra-15 centrifugal filter units (MWCO 30–100 kDa) to remove free lipids and residual ethanol. CAR mRNA–LNPs (2 µg of encapsulated mRNA per 2 × 10^5^ NK cells) were directly added to CB-derived NK cells. CB-derived NK cells were cultured in CTS NK-Xpander medium (Gibco) supplemented with 10% FBS and hIL-2 (100 IU/mL). NK cells were mixed thoroughly with the mRNA–LNPs and incubated overnight in CTS NK-Xpander medium with 10% FBS and 100 IU/mL hIL-2. At 16–24 h post transfection, CAR-NK cells were harvested either for subsequent analysis or for downstream applications.

### Lentiviral transduction and generation of stable cell lines

Lentiviral particles were produced in 293 T cells by co-transfection of transfer plasmids together with the packaging plasmids psPAX2 and pMD2G. Viral supernatants were collected at 48 h and 72 h post-transfection, filtered through a 0.45 μm membrane, and used for transduction of target cells in the presence of 8 μg/mL polybrene.

To generate CLL-1–overexpressing Nalm-6 cells, a lentiviral vector encoding human CLL-1 was used to transduce Nalm-6 cells. Cells were centrifuged with viral supernatant at 1200 g for 90 min to enhance infection efficiency and cultured for 48 h, followed by puromycin selection. Surface CLL-1 expression was confirmed by flow cytometry.

To establish luciferase–mCherry–expressing THP-1 cells, THP-1 cells were first transduced with a luciferase–mCherry lentiviral construct. Fluorescent cells were sorted by fluorescence-activated cell sorting (FACS) to obtain a stable THP-1–Luc^+^ cell line. For generation of HLA-E–overexpressing cells, THP-1–Luc^+^ cells were subsequently transduced with an HLA-E–encoding lentiviral vector, followed by puromycin selection and validation of HLA-E surface expression by flow cytometry, yielding THP-1–Luc^+^–HLA-E cells [44].

For JAK2 knockdown, lentiviral particles expressing scramble control or JAK2-targeting shRNA were produced as described above and used to transduce THP-1 and OCI-AML2 cells. Stable knockdown cells were selected and validated by Western blot analysis confirming reduced JAK2 protein expression.

### Generation of CLL-1 CAR-T Cells via lentiviral transduction

CAR-T cells were generated by lentiviral transduction. Peripheral blood mononuclear cells were isolated from healthy donors, and CD3^+^ T cells were enriched using EasySep™ Human T Cell Isolation Kit (StemCell). The T cells were activated using CD3/CD28 Dynabeads (Gibco) and cultured in complete medium supplemented with 100 IU/mL hIL-2 (PeproTech). After 72 h of activation, cells were transduced with lentiviral supernatant encoding the CAR construct at a multiplicity of infection (MOI) of 10. Cells were seeded at 5 × 10^5^ per well in 24-well plates and centrifuged at 1800 × g for 60 min to enhance transduction efficiency.

Following infection, cells were maintained in IL-2–containing medium. Transduction efficiency was assessed 3–5 days later by flow cytometric analysis of CAR expression. Cells were subsequently expanded to the required numbers, and CAR expression was verified prior to each functional assay.

### Western blot analysis

Cells were lysed in RIPA buffer supplemented with protease and phosphatase inhibitors. Protein concentration was quantified using the BCA assay. Equal amounts of protein were separated by SDS-PAGE and transferred to PVDF membranes. Membranes were blocked in 5% non-fat milk and incubated with primary antibodies against JAK2, p-JAK2, STAT1, p-STAT1, HLA-E, or GAPDH. Following incubation with HRP-conjugated secondary antibodies, signals were detected using ECL chemiluminescence and imaged with the Amersham Imager 600. Densitometry was performed with ImageJ, and protein expression was normalized to GAPDH.

### Cytotoxicity assay

CFSE- or CellTrace-Violet-labeled (Invitrogen) target cells were co-cultured with CLL-1 CAR-T, mock T, CLL-1 CAR-NK or NT-NK cells at the indicated E:T ratios in triplicate for the indicated times in RPMI 1640 media supplemented with 10% FBS. Total cells were harvested and then labeled with Annexin V-APC or Annexin V-FITC (both Biolegend) for flow cytometry analysis.

### IFN-γ and Granzyme B secretion assay by ELISA

Cytokine levels were quantified using sandwich ELISA kits (Dayou, Dakewe) according to the manufacturer’s instructions. Briefly, diluted standards, samples, and blank controls (100 μL per well) were added to 96-well plates in duplicate. After incubation with biotinylated detection antibody (50 μL per well, 2 h, room temperature), the wells were washed three times with 1 × Washing Buffer and then incubated with Streptavidin-HRP solution (100 μL per well, 20 min, room temperature). Following an additional washing step, color development was performed using TMB substrate (100 μL per well, 10–20 min, room temperature, dark). The reaction was terminated with Stop Solution (100 μL per well), and absorbance was measured at 450 nm with a reference wavelength of 610–630 nm.

### Colony-forming cell assay

A total of 5 × 10^6^ PBMCs from healthy donors were either co-cultured with non-transfected NK cells, CLL-1 CAR-NK cells, or media alone at an E:T ratio of 1:1 for 24 h, or treated with 2 μM venetoclax for 24 h. Then, CD34^+^ cells were sorted by flow cytometry. Cells (500 per well) were plated in 96-well plates and treated with HemaTox Myeloid Medium (StemCell Technologies, USA) in triplicate. After 7 days of culture for myeloid differentiation, absolute cell counts were determined using precision count beads (Biolegend) on a LSRFortessa X-20 flow cytometer (BD Biosciences, USA). Cell differentiation was assessed in triplicate through staining of cells using anti-CD13-APC, anti-CD14-FITC and anti-CD15-PE (all Biolegend) for myeloid lineage. For colony-forming cell assay on CD34^+^ cells, MethoCult H4535 (StemCell Technologies, USA) was used according to the manufacturer’s instructions. Briefly, sorted CD34^+^ cells were seeded on 6-well plates (1000 cells per well) in triplicate in 1 mL methylcellulose medium. After 14 days, CFU-G, CFU-M, and CFU-GM colonies were enumerated.

### RNA sequencing (RNA-seq)

For bulk RNA sequencing analysis, tumor cells were isolated from co-culture mixture by flowcytometry cell sorting system. Total RNA was extracted using the TRIzol reagent (Invitrogen, CA, USA) according to the manufacturer’s protocol. RNA purity and quantification were evaluated using the NanoDrop 2000 spectrophotometer (Thermo Scientific, USA). RNA integrity was assessed using the Agilent 2100 Bioanalyzer (Agilent Technologies, Santa Clara, CA, USA). Then the libraries were constructed using VAHTS Universal V10 RNA-seq Library Prep Kit (Premixed Version) according to the manufacturer’s instructions. The libraries were sequenced on an Illumina Novaseq 6000 platform and 150 bp paired-end reads were generated. The transcriptome sequencing and analysis were conducted by OE Biotech Co., Ltd. (Shanghai, China). Differentially expressed gene analysis was performed with the R package DESeq2 with p value < 0.01 and fold change > 1 as thresholds. Pathway enrichment of DEGs was analyzed by gene ontology (GO) and hallmark gene sets.

### In vivo experiments with mouse leukemia models

Female NOD.Cg-*Prkdc*^*scid*^*Il2rg*^*em1Smoc*^ (NSG) mice, aged 6–8 weeks, were purchased from Shanghai Model Organisms Center, Inc. Luciferase-mCherry^+^-expressing THP-1 cells (THP-1–Luc^+^, 5 × 10^5^) were injected intravenously (i.v.) into NSG mice. Beginning on day 2, mice received i.v. injections of PBS, non-treated NK cells (5 × 10^6^), or CLL-1 CAR-NK cells (5 × 10^6^) on days 2, 4, 6, and 8, for a total of four infusions. CAR mRNA–LNP transfection of NK cells was performed one day before infusion to ensure optimal CAR expression at the time of administration. Peripheral blood was collected every other day starting from the day after the final infusion, for a total of four time points, to assess the persistence of infused NK cells in the CAR-NK group, using precision count beads (Biolegend) followed by the manufacturer’s instructions. Serum cytokines, including GM-CSF and IFN-γ, were quantified by ELISA (Dayou, Dakewe) on day 1 following the first CAR-NK cell infusion. To assess HLA-E expression on residual tumor cells after CAR-NK cell administration, mice were injected with THP-1–Luc^+^ cells, followed by CAR-NK cell infusion the next day. Two days later, bone marrow samples were collected and, after red blood cell lysis, cells were stained for HLA-E and analyzed by flow cytometry to determine the surface HLA-E MFI on mCherry^+^ tumor cells. In the THP-1–Luc^+^–HLA-E xenograft model, mice received CAR-NK cell infusions on days 2, 4, 6, and 8. Mice were additionally treated with either PBS, or CAR-NK cells combined with IgG4 isotype control (80 μg/mouse, MCE) or monalizumab (80 μg/mouse, MCE), administered on days 2, 4, and 8. All animals were housed under specific pathogen-free (SPF) conditions in accordance with institutional guidelines, and all experimental procedures were approved by the Institutional Animal Care and Use Committee (IACUC, JUMC2025-286-B).

### Statistical analysis

All statistical analyses and graphical representations were conducted using R (v4.4.2) and GraphPad Prism (v10.2.0). For all statistical tests, **** refers to *p* < 0.0001, *** refers to *p* < 0.001, ** refers to *p* < 0.01 and * refers to *p* < 0.05.

## Results

### CLL-1 Expression Is Elevated in MDR AML patients

**Fig. 1:**
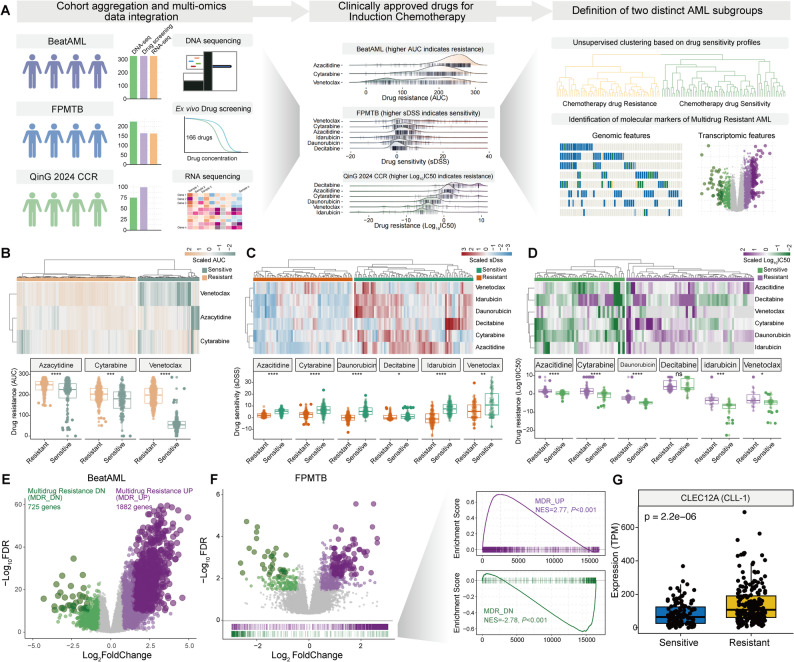
Identification of CLL-1 as a selective AML antigen upregulated in multidrug resistant (MDR) cases. **A** Schematic of ex vivo drug response analysis in three independent AML cohorts (FPMTB, BeatAML, and Qin et al.), focusing on key first-line agents including cytarabine, daunorubicin, venetoclax, azacitidine, idarubicin, and decitabine. **B**–**D** Unsupervised clustering of drug-sensitivity profiles stratified patients into two major pharmacological subgroups: multidrug-sensitive AML and MDR AML. Heatmaps depict clustering results across cohorts, with MDR cases characterized by broad resistance to multiple agents. **E** Transcriptomic profiling of BeatAML samples identified 2,607 differentially expressed genes (DEGs) between MDR and sensitive groups, including 1,882 upregulated genes (MDR_UP) and 725 downregulated genes (MDR_DN). **F** Validation of MDR gene signatures in the FPMTB cohort using gene set enrichment analysis (GSEA). **G** Comparative analysis of clinically investigated AML CAR targets revealed selective upregulation of CLL-1 in MDR AML. Mann–Whitney U test. Data represent integrated analyses of drug sensitivity (sDSS, AUC, and IC50) and transcriptomic profiles across cohorts, with DEGs defined by FDR < 0.05 and |log_2_ fold change|> 0.8

To evaluate ex vivo responses to first-line therapies in AML, we analyzed drug screening datasets from three independent cohorts, focusing on key agents such as cytarabine, daunorubicin, venetoclax, azacitidine, idarubicin and decitabine (Fig. 1A). Unsupervised clustering of drug sensitivity profiles revealed two major pharmacological subgroups: a multidrug-sensitive AML group and an MDR AML group, characterized by global responsiveness or resistance across multiple agents, respectively (Figs. 1B–1D).

To investigate transcriptomic differences between these groups, we integrated RNA-seq data from the BeatAML cohort. Comparative analysis identified 2,607 differentially expressed genes (DEGs), including 1,882 upregulated genes in MDR-AMLs, hereafter referred to as the MDR_UP signature (Fig. 1E, Table S1), and 725 downregulated genes, designated as the MDR_DN signature (Fig. 1E). We next validated these gene signatures using gene set enrichment analysis (GSEA) in the FPMTB cohort. Consistent with expectations, MDR_UP genes were positively enriched in the MDR-resistant AML subgroup, whereas MDR_DN genes were negatively enriched (Fig. 1F). Importantly, comparative assessment of clinically investigated CAR targets in AML revealed that only CLL-1 was included in the MDR_UP genes, exhibiting significantly elevated expression in Resistant-AMLs (Fig. 1G). In contrast, the expression levels of CD33, CD7, and CD38 remained largely unchanged, whereas CD123 (IL3RA) expression was downregulated (Figure S1A). Collectively, these findings delineate CLL-1 as the uniquely currently validated AML antigen selectively upregulated in MDR cases, emphasizing its potential translational significance as a therapeutic target in this clinically challenging subgroup.

### Efficient generation of primary CLL-1 CAR-NK cells via CAR mRNA–LNP transfection

**Fig. 2:**
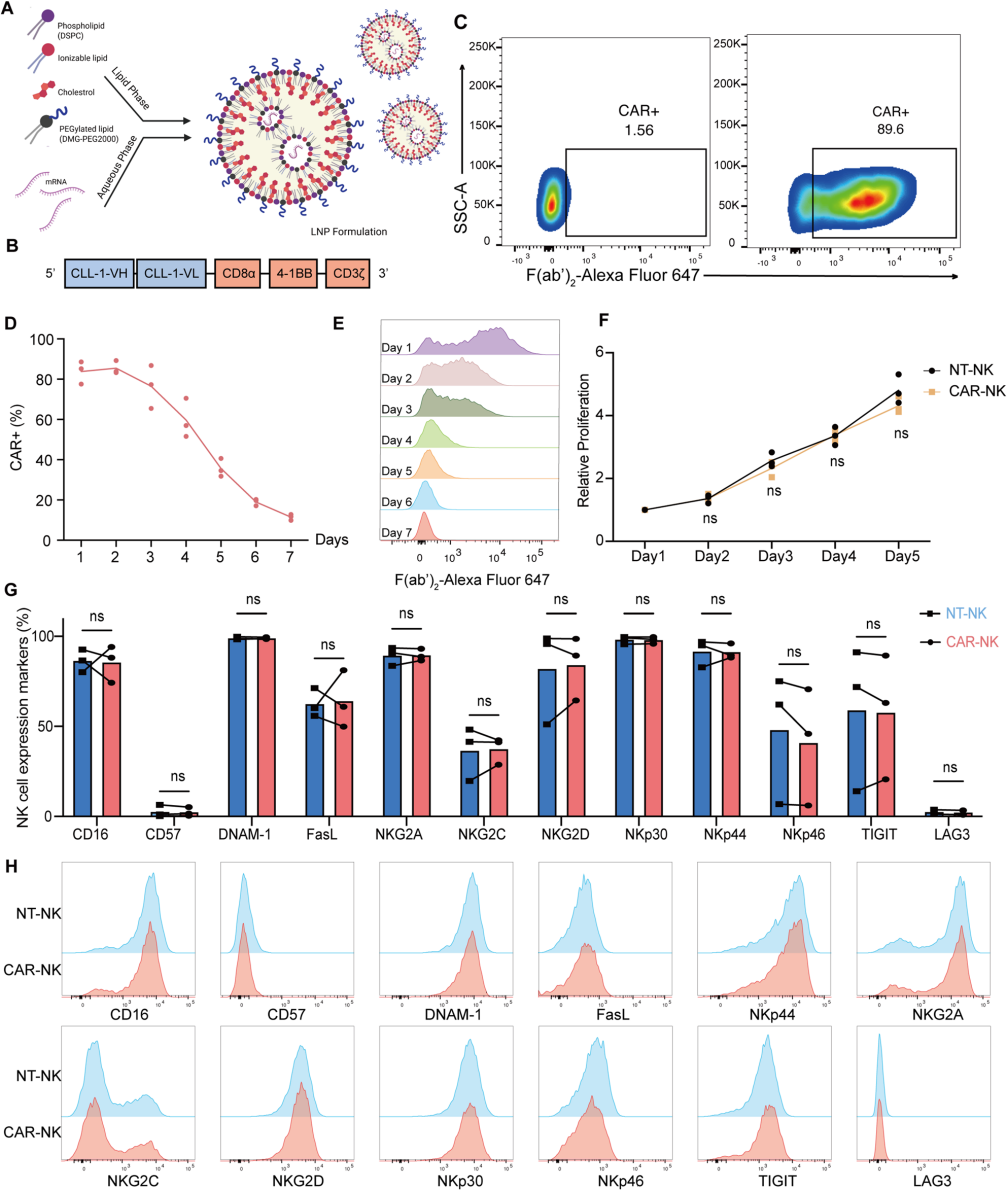
Development of mRNA–LNP-transfected CLL-1 CAR-NK cells. **A** Graphic representation of the workflow for CLL-1 CAR mRNA-LNP generation. **B** Schematic of the CLL-1 CAR vector containing the anti-human CLL-1 scFv linked to CD8α, 4-1BB costimulatory domains, and CD3ζ signaling domain. **C** Flow cytometry was performed using an F(ab’)_2_ antibody recognizing the humanized CLL-1 CAR construct. NK cells transfected with CLL-1 CAR were gated based on fluorescence signals relative to non-transfected NK cell controls. **D**–**E** The frequency of CLL-1 CAR-expressing from 3 independent CB donors-derived NK cells was monitored from day 1 to day 7 post-transfection (*n* = 3), and representative histograms are shown. **F** Proliferation of CLL-1 CAR-NK verses NT-NK cells during long-term culture (*n* = 3). Two-way ANOVA multiple comparisons. **G** Expression of NK cell receptors analyzed by flow cytometry after 24 h of culture (*n* = 3). paired t-test. (H) Representative flow cytometry histogram showing mean fluorescence intensity (MFI) of NK cell-specific markers in CLL-1 CAR-NK and NT-NK cells (data from one representative donor). Data are presented as mean ± SD. ns, *p* > 0.05

LNPs were successfully formulated by microfluidic mixing of a lipid solution and an aqueous CAR mRNA solution. The lipid mixture (ionizable lipid, cholesterol, DSPC, and PEG2000 at a molar ratio of 50:38.5:10:1.5) was dissolved in 90% ethanol and 10% citrate buffer (pH 4), whereas the aqueous phase contained base-modified CAR mRNA in citrate buffer (pH 4). Controlled mixing at 10 ml/min with a 2:1 volumetric ratio (mRNA:lipid) yielded LNPs at a final mRNA concentration of 0.5 mg/ml (Fig. 2A). The CAR construct comprised a CLL-1–specific scFv, a 4-1BB costimulatory domain, and a CD3ζ signaling chain (Fig. 2B).

Ex vivo expansion of CB-derived NK cells generated a CD3^−^CD56^+^ population with a purity of up to 95% (Figure S1B). Direct LNP-mediated transfection resulted in dose-dependent CAR expression, reaching peak efficiency of nearly 90% (Fig. 2C). Expression was maintained for up to 48 h following 16-24 h of transfection without compromising cell proliferation and viability (Figs. 2D–2F, S1C). Importantly, the expression of key activating markers (CD16, CD57, DNAM-1, FasL, NKG2C, NKG2D, NKp30, NKp44, NKp46) and inhibitory receptors (NKG2A, TIGIT, LAG3) remained comparable between CAR-NK and NT-NK cells (Figs. 2G–2H).

Together, these results establish the feasibility of generating primary CLL-1 CAR-NK cells using an mRNA–LNP platform, achieving high transfection efficiency while preserving NK cell viability and phenotype.

### CLL-1 CAR-NK cells exhibit potent antitumor activity with limited off-target toxicity to HSC

**Fig. 3:**
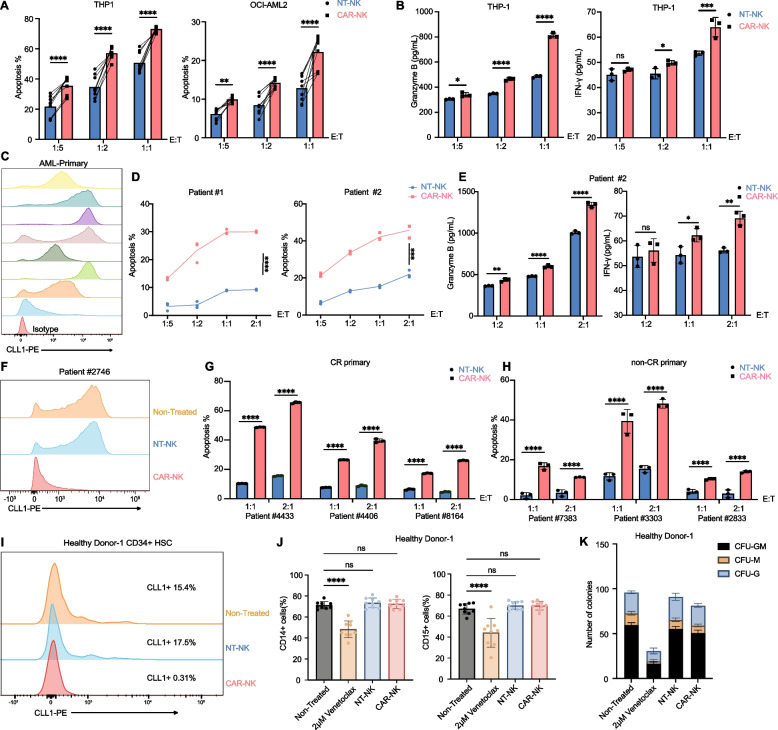
Expression of CLL-1 and functional activity of CLL-1 CAR-NK cells. **A** Different CB-derived CLL-1 CAR-NK cells lysed CLL-1^+^ cell lines THP-1 and OCI-AML2 at different E:T ratios (*n* = 9). E:T, effector-to-target cell ratio. **B** CLL-1 CAR-NK cells secreted IFN-γ and Granzyme B in a dose-dependent manner against THP-1 cells, measured by ELISA. **C** Expression of CLL-1 on eight primary AML samples. **D** Apoptosis of thawed primary bone marrow (BM)-derived cells from two AML patients who were ineligible for intensive chemotherapy or Ven/Aza treatment after 4 h co-culture with NT-NK or CLL-1 CAR-NK cells at different E:T ratios (*n* = 3). **E** IFN-γ and Granzyme B secretion by NT-NK or CLL-1 CAR-NK cells against patient #2 target cells, measured by ELISA (*n* = 3). **F** Expression of CLL-1 in primary cells of patient #2746 after 4 h co-culture with CLL-1 CAR-NK cells, NT-NK cells, or medium. **G** Apoptosis of BM-derived cells from three AML patients who were eligible for intensive or targeted therapy after 4 h co-culture with NT-NK or CLL-1 CAR-NK cells at different E:T ratios (*n* = 3). **H** Apoptosis of BM-derived cells from three non-CR AML patients after first-cycle therapy following 4 h co-culture with NT-NK or CLL-1 CAR-NK cells at different E:T ratios. (*n* = 3). **I** Expression of CLL-1 in healthy donor CD34^+^ hematopoietic stem cells (HSCs) after 4-h co-culture with CLL-1 CAR-NK cells, medium, or NT-NK cells. **J**-**K** PBMCs from healthy donor-1 were co-cultured with either NT-NK cells, CLL-1 CAR-NK cells, or medium alone at an E:T ratio of 1:1 for 24 h, or with 2 μM venetoclax for 24 h. CD34^+^ cells were then sorted by FACS and plated either in HemaTox myeloid medium for 7 days, followed by flow cytometric quantification of CD14^+^ and CD15.^+^ cells (J; *n* = 9), or in semisolid methylcellulose-based growth medium for 14 days, followed by colony counting of CFU-G, CFU-M, and CFU-GM colonies (K; *n* = 9). Data are shown as mean ± SD from at least three independent experiments. Two-way ANOVA multiple comparisons. ****p < 0.0001, ***p < 0.001, **p < 0.01, *p < 0.05, ns *p* > 0.05

CLL-1 has been reported to be expressed on myeloid cells and leukemic blasts in AML [45]. To evaluate whether CLL-1 represents an ideal target for CAR-NK therapy in AML, we first assessed its surface expression on AML cell lines and primary AML blasts by flow cytometry. As shown, the AML cell lines THP-1, OCI-AML2, and HL-60 exhibited variable levels of CLL-1 expression, whereas the acute lymphoblastic leukemia cell line Nalm-6 was negative (Figures S2A–S2B). In 4-h co-culture assays, CLL-1 CAR-NK cells exhibited potent cytotoxicity against THP-1 and HL-60 cells even at low E:T ratios, and demonstrated enhanced killing of OCI-AML2 cells, which are typically resistant to NK-mediated cytotoxicity (Figs. 3A and S2C) [46]. In contrast, no increased cytotoxicity was observed against Nalm-6 cells (Figure S2D). However, overexpression of CLL-1restored susceptibility to killing, confirming the antigen specificity of CLL-1 CAR-NK cells (Figure S2D). ELISA analysis of supernatants revealed that CAR-NK cells secreted higher levels of granzyme B and IFN-γ compared with NT-NK cells (Figs. 3B and S2E).

Bone marrow samples from eight AML patients confirmed detectable CLL-1 expression in most cases (Fig. 3C). In primary blasts from patients not eligible for intensive therapy, NT-NK cells induced minimal lysis, whereas CAR-NK cells exhibited markedly enhanced killing (Fig. 3D), accompanied by increased granzyme B and IFN-γ levels (Figs. 3E and S2F). Similarly, in patients who were either eligible for intensive or targeted therapy and capable of achieving complete remission (CR-primary), or refractory to prior treatments (non-CR primary), CAR-NK cells exhibited potent and selective cytolysis of primary AML blasts and efficiently eradicated CLL-1^+^ cells (Figs. 3F–3H). In addition, we compared the efficacy of mRNA-LNP-transfected CLL-1 CAR-NK cells with lentiviral-transduced CLL-1 CAR-T cells (Figure S2G). At the same E:T ratio, CAR-T cells showed lower cytotoxicity than CAR-NK cells after 4 h of co-culture (Figure S2H). However, during prolonged killing assays (48 and 72 h), CAR-T cells exhibited stronger cytotoxic activity (Figure S2I–S2J), likely reflecting their sustained proliferative capacity and persistence, which enables progressive accumulation of effector function over time.

To evaluate potential off-target toxicity, we examined the impact of CLL-1 CAR-NK cells on normal HSCs. CAR-NK cells were co-cultured with PBMC from healthy donors. At a 1:1 E:T ratio after 4 h, CD34^+^CLL-1^+^ cells were efficiently eliminated (Fig. 3I). However, long-term culture in HemaTox medium or methylcellulose revealed no significant reduction in lineage-specific progenitors (CD13^+^, CD14^+^ or CD15^+^) (Figs. 3J and S3A–S3C) or colony forming unit (CFU) output (CFU-GM, CFU-G, CFU-M) (Figs. 3K and S3D) relative to medium-only or NT-NK controls. By contrast, treatment with 2 μM venetoclax for 24 h (mimicking clinically relevant plasma concentrations) resulted in marked toxicity to HSCs (Fig. 3J–3K, and S3A–S3C). [47] Collectively, these findings indicate that mRNA–LNP–transfected CLL-1 CAR-NK cells can be safely applied for AML therapy without inducing HSC-targeted toxicity.

### Repeated infusions of mRNA–LNP CLL-1 CAR-NK cells suppress leukemia progression and prolong survival in vivo

**Fig. 4:**
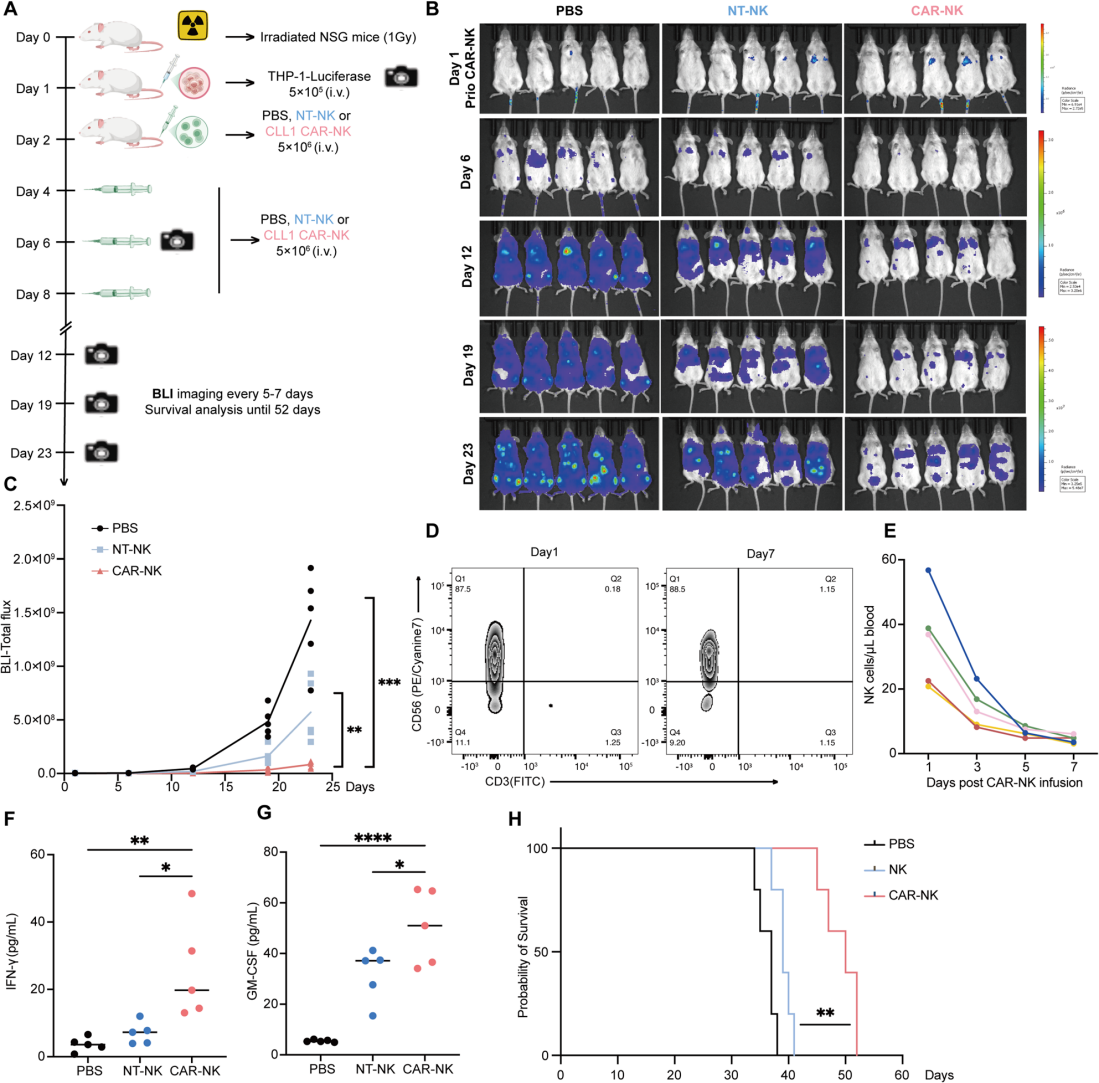
Repeated infusions of mRNA–LNP CLL-1 CAR-NK cells suppress leukemia progression and prolong survival in THP-1 xenograft models. **A** Schematic representation of the in vivo experimental layout: NSG mice were inoculated with 5 × 10^5^ THP-1-Luc^+^ cells. On days 2, 4, 6, and 8, mice were injected i.v. with a single dose of 5 × 10^6^ NT-NK or CLL-1 CAR-NK cells or PBS, followed by bioluminescence imaging (BLI) every 5–7 days until day 23. **B** Representative BLI images of PBS-, NT-NK-, or CLL-1 CAR-NK-treated mice at days 6, 12, 19, and 23 (*n* = 5). **C** Quantification of BLI total flux at each time point. one-way ANOVA. **D**–**E** Peripheral blood was collected from mice following the fourth infusion of CAR-NK cells. Human NK cells (hCD45^+^CD3^−^CD56^+^) were quantified by flow cytometry using precision counting beads to determine absolute cell numbers in vivo (*n* = 5). **F**–**G** Serum cytokine analysis of peripheral blood on day 1 post first NK cell injection, showing increased GM-CSF and IFN-γ levels in CLL-1 CAR-NK–treated mice (*n* = 5). one-way ANOVA. **H** Kaplan–Meier survival analysis of mice treated with PBS, NT-NK, or CLL-1 CAR-NK cells (*n* = 5). Log-rank test. Mean ± SD. ****p < 0.0001, ***p < 0.001, **p < 0.01, *p < 0.05, ns *p* > 0.05; Panel A was created with BioGDP

To investigate the in vivo efficacy of CLL-1 CAR-NK cells, luciferase-mCherry-expressing THP-1 (THP-1–Luc^+^) cells were injected intravenously into irradiated (1 Gy) NOD.Cg-*Prkdc*^*scid*^*Il2rg*^*em1Smoc*^ (NSG) mice. After confirming uniform engraftment by bioluminescence imaging (BLI), mice received four doses of either CAR-NK or NT-NK cells every other day, with PBS-injected mice serving as untreated controls (Fig. 4A). NT-NK treatment modestly delayed leukemia progression compared with PBS, whereas CLL-1 CAR-NK treatment resulted in profound leukemia reduction on days 12, 19, and 23 after THP-1 injection (Figs. 4B–4C). Peripheral blood monitoring showed that although NK purity remained stable, absolute NK cell counts declined by ~ 50% beginning on day 3 in CAR-NK group after the fourth infusion (Fig. 4D–4E). Cytokine analysis after the first infusion showed significantly elevated IFN-γ and GM-CSF levels in the CAR-NK group compared with NT-NK or PBS controls (Fig. 4F–4G). Importantly, mice treated with CLL-1 CAR-NK cells exhibited significantly prolonged survival relative to NT-NK–treated animals (Fig. 4H). Together, both ex vivo and in vivo experiments demonstrate that CLL-1 CAR-NK cells effectively eliminate AML cells and exert transient yet potent antitumor activity in vivo.

### Inflammatory Activation and HLA-E–Mediated Evasion Limit CAR-NK Function, Rescued by NKG2A Blockade

Given the transient expression of mRNA–LNP–transfected CLL-1 CAR-NK cells, together with the short-lived persistence of NK cells, repeated infusions are typically required to maintain antitumor activity. We hypothesized that sustained exposure to CAR-NK cells may impose selective pressure on tumor cells, thereby inducing adaptive changes that limit subsequent NK-cell function. To investigate tumor-intrinsic mechanisms associated with prolonged CAR-NK exposure, we performed transcriptomic analysis of leukemia cells that survived extended co-culture.

**Fig. 5:**
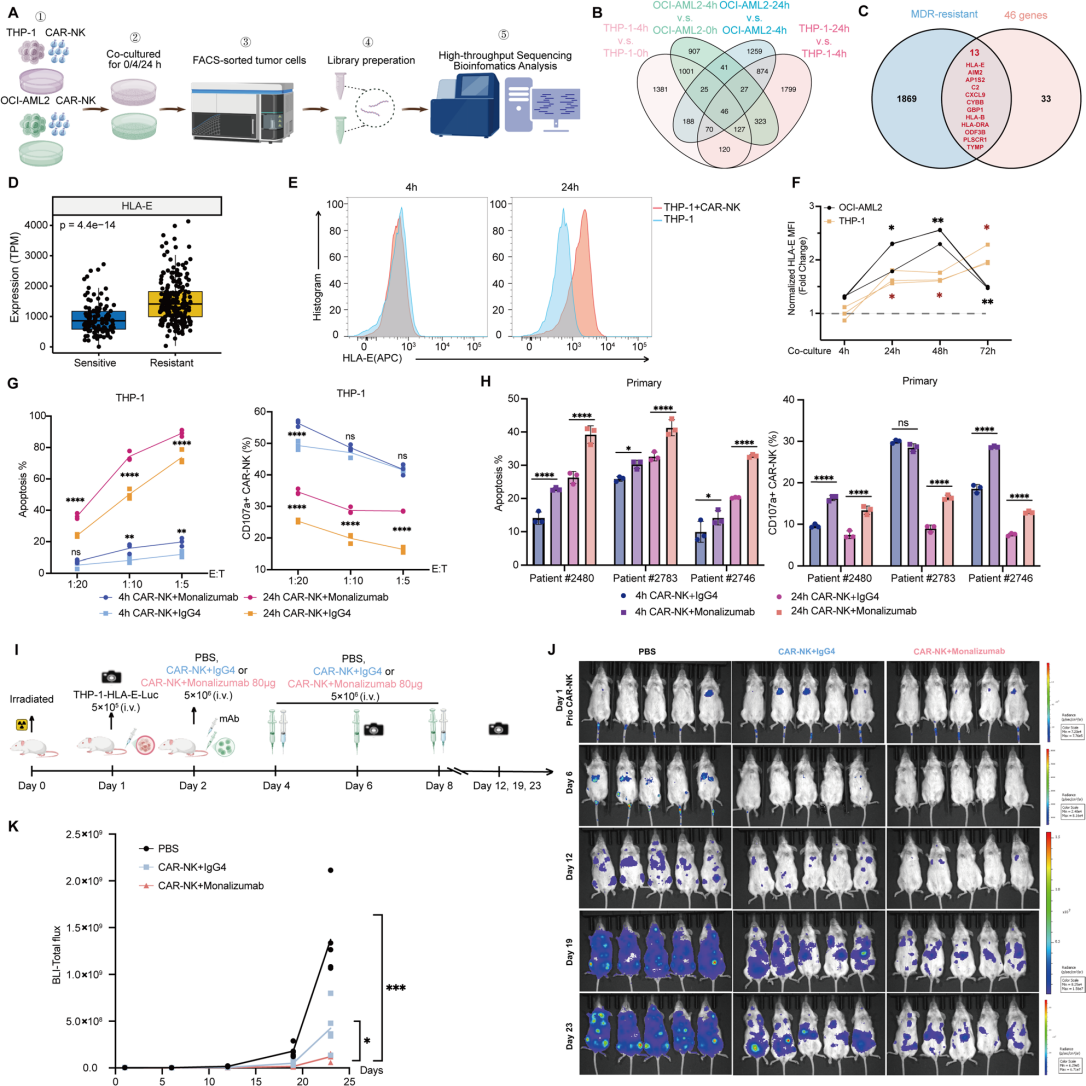
HLA-E-mediated immune evasion during prolonged CAR-NK cell engagement in AML cell lines and primary cells. **A** Schematic representation of RNA-seq analysis: THP-1 (E:T = 1:5) and OCI-AML2 (E:T = 1:1) cells were co-cultured with CLL-1 CAR-NK cells for 0, 4, or 24 h, and surviving tumor cells were sorted by FACS for transcriptomic profiling (*n* = 3). **B** Venn diagram illustrating 46 genes progressively upregulated over time in both THP-1 and OCI-AML2 cells. **C** Intersection of the 46 overlapping genes with the MDR_UP gene set (Fig. 1, Table S1), yielding 13 candidate genes. **D** Differential expression of HLA-E in MDR patients versus multidrug-sensitive AML samples. Mean ± SD; Mann–Whitney U test. **E** Representative flow cytometry histograms of HLA-E surface expression on THP-1 cells cultured alone or with CAR-NK cells for 4 or 24 h. E:T = 1:5. **F** Surface expression of HLA-E on THP-1 (E:T = 1:5) and OCI-AML2 (E:T = 1:1) cells co-cultured with CLL-1 CAR-NK cells or treated with IFN-γ (800 U/mL) for 4 h, 24 h, 48 h, and 72 h (*n* = 3). **G** Flow cytometric analysis of apoptosis of THP-1 cells and percentage of CD107a^+^ CAR-NK cells after 4 or 24 h co-culture with CAR-NK cells in combination with IgG4 isotype control or the anti-NKG2A monoclonal antibody Monalizumab (5 μg/mL) (*n* = 3). **H** Flow cytometric analysis of CD107a^+^ CAR-NK cells and apoptosis of bone marrow cells from three non-CR AML patients, following 4- or 24-h co-culture with CAR-NK cells (E:T = 1:2) in the presence of either an IgG4 isotype control or Monalizumab. (*n* = 3). **I** Schematic of the in vivo experimental design: NSG mice were inoculated with 5 × 10^5^ THP-1–Luc^+^–HLA-E cells. On days 2, 4, 6, and 8, mice were treated with four infusions of PBS, or CAR-NK cells combined with three doses of IgG4 isotype control or monalizumab administered on days 2, 4, and 8, followed by BLI every 5–7 days until day 23. **J** Representative BLI of mice treated with PBS, CAR-NK cells, or CAR-NK cells in combination with monalizumab at days 6, 12, 19, and 23 (*n* = 5). **K** Quantification of total flux from BLI analysis at each time point (*n* = 5). Two-way ANOVA for multiple comparisons. Mean ± SD; ****p < 0.0001, ***p < 0.001, **p < 0.01, *p < 0.05, ns *p* > 0.05. Panel A and I were created with BioGDP

THP-1 and OCI-AML2 cells were co-cultured with CAR-NK cells for 0, 4, and 24 h. Surviving tumor cells were sorted by flow cytometry and subjected to RNA-seq (Fig. 5A). Across both cell lines, 46 genes were identified as progressively upregulated over time (Fig. 5B, Table S2), and enrichment analysis indicated that these genes were mainly involved in antigen presentation and inflammatory response pathways (Figures S4A–S4B). Intersecting these genes with the previously identified MDR_UP gene set yielded 13 candidate genes (Fig. 5C). Among them, HLA-E is known to interact with the NK-cell inhibitory receptor NKG2A, for which blocking antibodies are available for therapeutic targeting, whereas the remaining candidates currently lack clearly actionable pharmacological interventions. Accordingly, we focused further analyses on the HLA-E–related pathway. Consistent with its inclusion in the MDR-associated dataset, HLA-E expression was higher in MDR AML patients than in multidrug-sensitive patients (Fig. 5D).

In vitro, compared with 4 h of CAR-NK co-culture, HLA-E expression in AML cell lines and primary AML blasts was significantly increased after 24 h and remained elevated for 2–3 days (Figs. 5E–5F and S4C–S4D), suggesting that CAR-NK exposure may induce HLA-E upregulation in tumor cells. We next assessed whether this phenomenon also occurs in vivo. In a THP-1–Luc^+^ leukemia mouse model, CAR-NK cells were infused on day 2 after intravenous tumor inoculation on day 1, and bone marrow samples were collected on day 4 to analyze residual leukemia cells. Flow cytometry showed that, compared with controls, alive tumor cells from CAR-NK–treated mice displayed significantly increased HLA-E expression, indicating that CAR-NK therapy can induce adaptive upregulation of HLA-E in residual leukemia cells in vivo (Figures S4E).

To determine whether blockade of this inhibitory pathway could help sustain mRNA-LNP transfected CAR-NK effector function, we evaluated the effect of the anti-NKG2A monoclonal antibody monalizumab. In co-culture systems with THP-1, OCI-AML2, and HL-60 cell lines as well as primary AML blasts, the proportion of CD107a^+^ CAR-NK cells declined at 24 h compared with 4 h across multiple E:T ratios, whereas addition of monalizumab partially restored CD107a expression and enhanced CAR-NK cytotoxic activity (Figs. 5G–5H and S4F–S4G).

We next evaluated the therapeutic significance of NKG2A blockade in vivo. A THP-1––Luc^+^–HLA-E xenograft model was established to mimic the elevated HLA-E status observed in residual leukemia cells following CAR-NK therapy. NSG mice were irradiated with 1 Gy and intravenously injected with THP-1–Luc^+^–HLA-E cells. After confirming comparable tumor burden by BLI, mice received PBS or CAR-NK infusions on days 2, 4, 6, and 8, together with IgG4 isotype control or monalizumab administered on days 2, 4, and 8 (Figs. 5I and S4H). The combination of CAR-NK cells with monalizumab further improved leukemia control and prolonged mouse survival (Figs. 5J–5K and S4I).

Taken together, these results indicate that CAR-NK therapy can induce adaptive upregulation of HLA-E in residual leukemia cells, which may contribute to limiting sustained NK-cell function through NKG2A signaling. Blockade of NKG2A partially alleviates this therapy-induced immune evasion, thereby helping to enhance the persistence of CAR-NK antitumor activity.

### IFN-γ–JAK2–STAT1 signaling promotes HLA-E–mediated resistance to CAR-NK cytotoxicity

**Fig. 6:**
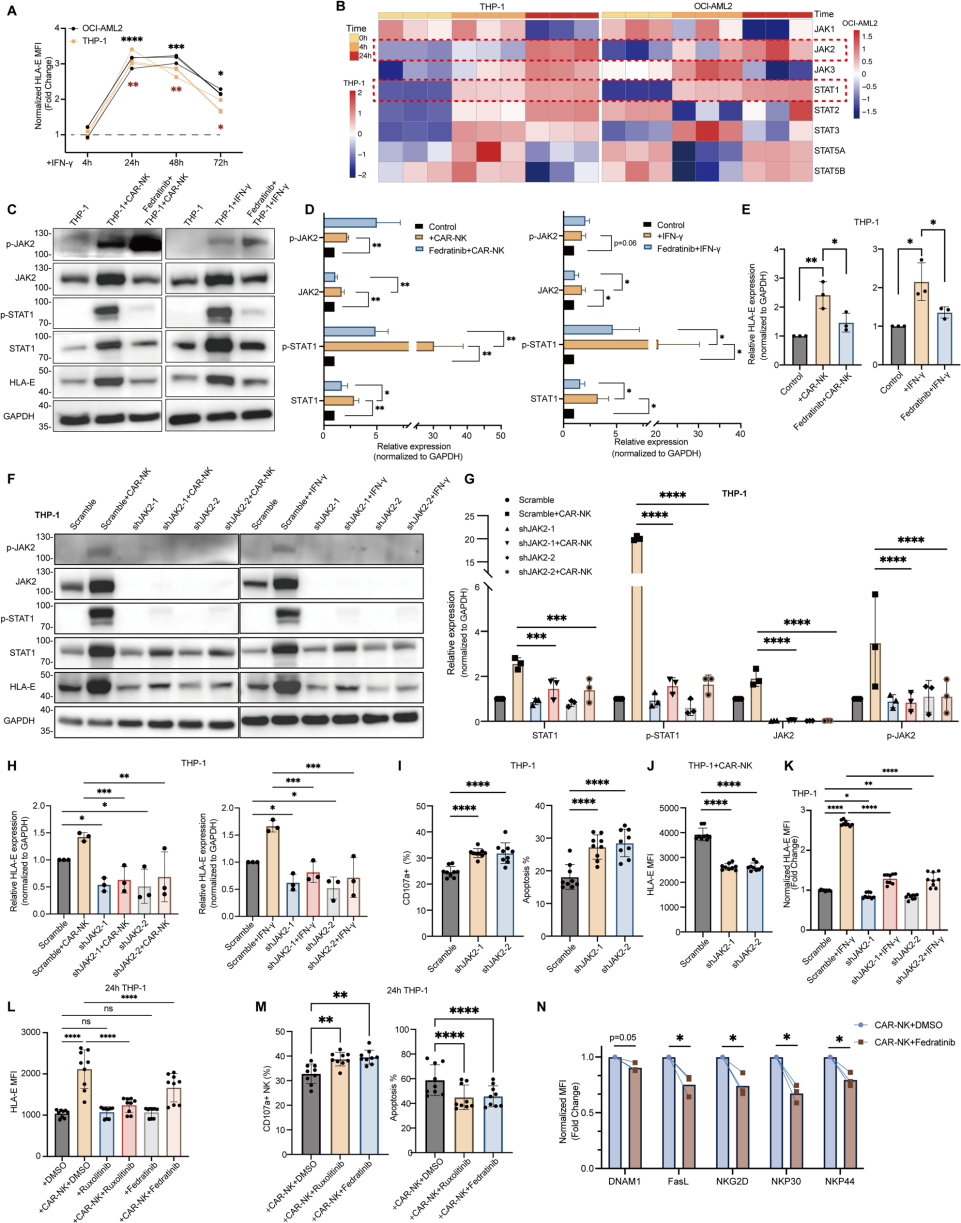
JAK2–STAT1 signaling contributes to HLA-E upregulation and effects CAR-NK cytotoxicity in AML cells. **A** Surface expression of HLA-E on THP-1 and OCI-AML2 cells treated with IFN-γ (800 U/mL) for 4 h, 24 h, 48 h, and 72 h (*n* = 3). **B** Heatmap of part of JAK-STAT family transcripts (JAK1, JAK2, JAK3, STAT1, STAT2, STAT3, STAT5A, STAT5B) in THP-1 and OCI-AML2 cells at 0 h, 4 h, and 24 h following CAR-NK co-culture. **C** Representative Western blots of JAK2–STAT1 pathway activation and HLA-E expression in THP-1 cells co-cultured with CAR-NK cells (E:T = 1:5) or treated with IFN-γ (800 U/mL) for 24 h in the presence or absence of Fedratinib (500 nM). **D**–**E** Densitometric quantification normalized to GAPDH of JAK2–STAT1 signaling (**D**) and HLA-E protein expression (**E**) from three independent experiments in THP-1 (*n* = 3). **F** Representative Western blots of JAK2–STAT1 pathway activation and total HLA-E expression in THP-1 cells expressing scramble control or JAK2 shRNA after 24 h of CAR-NK co-culture (E:T = 1:5) or IFN-γ treatment (800 U/mL). **G**–**H** Densitometric quantification normalized to GAPDH of JAK2–STAT1 signaling (**G**) and HLA-E expression (**H**) from three independent experiments in THP-1 (*n* = 3). **I** Flow cytometric analysis of CAR-NK CD107a degranulation and THP-1 apoptosis after 4 h co-culture with scramble or JAK2-knockdown cells (*n* = 9). E:T = 1:5. **J**–**K** Flow cytometry of surface HLA-E expression in scramble vs. JAK2-knockdown THP-1 cells following 24 h co-culture with CAR-NK cells (E:T = 1:5, J) or IFN-γ treatment (800 U/mL, K) (*n* = 9). **L** Comparison of HLA-E surface MFI in THP-1 cells after 24 h CAR-NK co-culture (E:T = 1:5) with or without the JAK inhibitors Ruxolitinib or Fedratinib (500 nM) (*n* = 9). (M) CAR-NK CD107a degranulation and THP-1 apoptosis after 24 h co-culture in the presence or absence of Ruxolitinib or Fedratinib (500 nM) (*n* = 9). **N** Expression of activating receptors on CAR-NK cells after 24 h culture with Fedratinib, normalized to DMSO-treated controls (500 nM) (*n* = 3). Two-way ANOVA for multiple comparisons. Data represent mean ± SD; statistical significance determined by two-way ANOVA for multiple comparisons. ****p < 0.0001, ***p < 0.001, **p < 0.01, *p < 0.05, ns *p* > 0.05

Since NKG2A blockade does not reduce HLA-E levels in tumor cells, we sought to directly suppress HLA-E expression. Transcriptomic analysis indicated the top-ranked hallmark pathway was the IFN-γ response (Figure S3B) prompting us to investigate the relationship between IFN-γ and HLA-E in AML. After prolonged culture, we observed that in both THP-1 and OCI-AML2 cells treated with IFN-γ, HLA-E expression began to increase by 24 h and remained upregulated for 2–3 days (Fig. 6A). IFN-γ typically exerts its biological effects by binding to broadly expressed cell-surface receptors and activating downstream JAK–STAT signaling pathways, thereby inducing transcriptional programs that regulate immune responses [48]. Consistent with findings in solid tumors, our transcriptomic results showed that only JAK2 and STAT1 remained elevated at 24 h, whereas JAK1 expression was reduced in AML (Fig. 6B and S5A) [49]. Thus, we hypothesized that the JAK2–STAT1 pathway may be involved in the upregulation of HLA-E in AML.

We therefore treated THP-1 and OCI-AML2 cells with Fedratinib, a selective JAK2 inhibitor, during 24-h CAR-NK co-culture or IFN-γ stimulation. After 24 h, western blot analysis showed increased total and phosphorylated JAK2 and STAT1, accompanied by a marked increase in HLA-E expression in both the co-culture and IFN-γ-treated groups (Fig. 6C–6E and S5B–S5D), while fedratinib effectively inhibited the JAK2–STAT1 pathway and reduced HLA-E magnitude (Fig. 6C, 6E, S5B, and S5D). To further validate this mechanism, we knocked down JAK2 in THP-1 and OCI-AML2 cells, which led to suppression of the JAK2–STAT1 pathway under both CAR-NK co-culture and IFN-γ treatment and reduced the magnitude of HLA-E induction (Fig. 6F–6H and S5E–S5I). In addition, compared with the scramble control, JAK2 knockdown also lowered baseline HLA-E expression (Fig. 6F, 6H, S5F, and S5I). We then examined whether JAK2 knockdown in AML cells could enhance CAR-NK cytotoxicity. Indeed, compared with scramble controls, JAK2-knock-down tumor cells showed significantly increased sensitivity to CAR-NK killing, higher proportions of CD107a^+^ CAR-NK cells, and reduced surface HLA-E expression after CAR-NK co-culture or IFN-γ stimulation (Fig. 6I–6K and S6A–S6C).

We next tested Fedratinib and the clinically used JAK2 inhibitor Ruxolitinib during CAR-NK co-culture or IFN-γ treatment. Both agents attenuated the 24-h increase in surface HLA-E expression induced by either CAR-NK co-culture or IFN-γ stimulation (Fig. 6L and S6D–S6E). However, neither drug lowered basal surface HLA-E expression, nor did they reduce HLA-E levels during a 4-h co-culture (Fig. 6L, S6D and S6F). Although both drugs increased the proportion of CD107a^+^ CAR-NK cells after 24 h, tumor apoptosis was unexpectedly reduced (Fig. 6M and S6G). A similar reduction in apoptosis was observed after 4 h of co-culture, despite no detectable change in the proportion of CD107a^+^ CAR-NK cells (Figure S6H–S6I). These observations prompted us to consider whether decreased apoptosis might result from a direct effect of the drugs on CAR-NK cells. After culturing CAR-NK cells with fedratinib for 24 h, we detected reduced expression of several activation markers, including DNAM-1, FasL, NKG2D, NKp30, and NKp44 (Fig. 6N and S6J). Collectively, these results indicate that although JAK2 inhibitors attenuate HLA-E induction in tumor cells, their direct inhibitory effects on CAR-NK activation ultimately diminish CAR-NK cytotoxicity, thereby limiting their therapeutic utility.

## Discussion

In recent years, CAR-T cell therapies have achieved remarkable clinical success in B-cell non-Hodgkin lymphoma (B-NHL) [50, 51]. However, their application in AML remains challenging due to limited efficacy, the paucity of selective targets, and frequent on-target, off-tumor toxicities [50, 52]. Current evidence suggests that CLL-1 and CD7 targeting approaches, particularly in the context of bridging transplantation, represent the most promising strategies to date for AML [53, 54]. Autologous CAR-T therapies, which are currently applied in the clinical setting, require the collection of patient-derived T cells and involve time-consuming and costly manufacturing processes [55–57]. Against these backdrops, our study focused on developing an mRNA–LNP platform to engineer CLL-1 CAR-NK cells, aiming to overcome key safety and efficacy barriers associated with existing CAR-T and CAR-NK approaches.

A major advantage of our strategy lies in the transient nature of CAR expression achieved through mRNA–LNP delivery. Despite the short duration of CAR expression, our platform consistently reached transfection efficiencies above 85%, while preserving NK cell viability and phenotype, consistent with a recent report [58]. In contrast to viral vector-based CAR approaches, which integrate transgenes into the genome and thus carry risks of insertional mutagenesis and long-term toxicity, mRNA transfection enables controlled and time-limited CAR expression [59–62]. This feature is particularly advantageous in AML, where most candidate antigens, including CLL-1, exhibit some expression on normal myeloid progenitors, raising safety concerns in the context of durable CAR activity [63]. Transient CAR expression minimizes the risk of prolonged hematopoietic toxicity while still allowing for meaningful antileukemic activity. Moreover, the inherently short in vivo persistence of NK cells complements the transient CAR expression profile, permitting repeated infusions that can be tailored to patient response, thereby allowing clinicians to optimize therapy intensity and duration while minimizing cumulative toxicity [64, 65]. Although mRNA–LNP delivery has been validated in clinical settings such as mRNA-based vaccines and is currently being explored in CAR-T therapies, its use in CAR-NK therapy remains confined to the preclinical stage, and clinical trials will be required to fully establish its therapeutic potential [58, 66, 67].

From a clinical standpoint, CAR-T and CAR-NK cell therapies are currently applied primarily in the treatment of relapsed or refractory hematologic malignancies, particularly in patients who have failed multiple prior regimens. [50, 51] The choice of CLL-1 as a CAR target is supported by both our preclinical data and prior clinical observations. Among multiple candidate AML targets such as CD33, CD123, CD7, and CD38, our analysis of MDR AML samples identified CLL-1 as a unique antigen consistently upregulated relative to drug-sensitive specimens, with stable expression across disease stages. Moreover, CLL-1 exhibits low-level expression on normal CD34⁺ HSCs, thereby reducing the risk of hematopoietic toxicity. Functionally, our mRNA–LNP–transfected CLL-1 CAR-NK cells exhibited potent cytolytic activity against AML cell lines in vitro and patient-derived leukemic blasts ex vivo, while sparing healthy donor HSCs. In xenograft mouse models, repeated administration of these CAR-NK cells significantly reduced leukemic burden and extended survival, confirming their therapeutic potential. Although the short duration of CAR expression may appear limiting for tumor elimination, our findings demonstrate that repeated administrations can still effectively augment antitumor efficacy, consistent with prior reports showing improved outcomes with multiple CAR-NK infusions. [68] Taken as a whole, the stable upregulation of CLL-1 in MDR AML, combined with its restricted expression on normal HSCs, underscores its promise as a therapeutic target for CAR-NK cell therapy in patients with refractory disease who have exhausted conventional treatment options.

To further enhance the therapeutic efficacy of CLL-1 CAR-NK cells, we investigated alterations in the tumor immune microenvironment following sustained cytotoxic pressure. Infusion of CLL-1 CAR-NK cells elicited a robust cytokine response, characterized by elevated IFN-γ and Granzyme B secretion, which contributed to tumor clearance but simultaneously induced rapid upregulation of HLA-E in AML cells. Furthermore, analysis of primary MDR AML samples confirmed elevated baseline HLA-E expression relative to sensitive cases. Engagement of the inhibitory receptor NKG2A by HLA-E impaired CAR-NK cytotoxicity, thereby limiting therapeutic effectiveness. [69, 70] Importantly, the observed increase in HLA-E expression appears secondary to immune activation. While NKG2A blockade alone has shown only modest benefit in late-stage solid tumors, likely due to the absence of an immune-activated microenvironment, its combination with CAR-NK cells may amplify immune activation and cytotoxic responses, representing a promising combinatorial strategy [71, 72].

Beyond receptor–ligand interactions, our data identify JAK2–STAT1 activation as a contributor to HLA-E induction in AML under CAR-NK or IFN-γ exposure. Transcriptomic and functional analyses consistently showed selective upregulation of JAK2 and STAT1, and both pharmacologic inhibition and JAK2 knockdown reduced HLA-E induction and partially restored CAR-NK cytotoxicity, indicating that the JAK2–STAT1 axis may act as an upstream link connecting cytokine pressure to HLA-E–mediated immune escape. However, given that JAK2 inhibition also impaired NK-cell activation and our primary focus was on strategies to decrease HLA-E and enhance CAR-NK efficacy, we did not further dissect the precise molecular mechanisms linking JAK2–STAT1 to HLA-E upregulation. This observation highlights a potentially important pathway but leaves detailed mechanistic exploration for future studies. Nevertheless, these findings reinforce the clinical relevance of directly targeting HLA-E or modulating the NKG2A/HLA-E axis to overcome adaptive resistance, emphasizing the therapeutic potential of combining CAR-NK therapy with checkpoint inhibition. The transient nature of mRNA–LNP–transfected CAR expression, together with repeated dosing, further supports strategies aimed at limiting HLA-E–mediated inhibition while preserving NK-cell function.

Taken together, our findings carry several clinical implications. First, the favorable safety profile of transient, non-integrating mRNA–LNP CAR-NK cells supports their application in heavily pretreated AML patients, for whom minimizing prolonged toxicities such as CRS and off-tumor effects is paramount. Second, the modularity of the mRNA–LNP platform facilitates rapid adaptation of CAR designs to alternative AML targets, enabling personalized approaches in the setting of disease heterogeneity. The LNP delivery approach used in this study currently lacks in vivo specificity for NK cells and is therefore better suited for adoptive transfer following ex vivo transient engineering. Given the transient nature of expression, repeated dosing may be required in clinical settings, which could be achieved either through multiple rounds of ex vivo transfection followed by sequential infusions, or by generating standardized batches of LNP-engineered NK cells for cryopreservation and subsequent on-demand administration. Previous studies have shown that repeated NK-cell infusions achieve superior therapeutic efficacy compared with a single infusion, and NK cells can retain high transgene expression after cryopreservation following transfection, supporting the translational feasibility of this strategy [58, 68]. Third, identification of the NKG2A/HLA-E axis and its upstream JAK2–STAT1 regulatory pathway as a mechanism of immune escape emphasizes the necessity of rational combinatorial strategies that integrate both antigen targeting and immune checkpoint modulation.

## Conclusion

Chemoresistance remains a major obstacle in AML therapy, and our integrative transcriptomic analysis identified CLL-1 as a consistently upregulated antigen in multidrug-resistant AML, underscoring its translational relevance. Using an mRNA–LNP delivery platform, we generated CLL-1 CAR-NK cells with high transfection efficiency, preserved viability, and potent antigen-specific cytotoxicity while sparing normal hematopoietic progenitors. Mechanistically, prolonged CAR-NK engagement induced HLA-E upregulation through activation of the JAK2–STAT1 pathway, promoting immune escape; importantly, this adaptive resistance was effectively reversed by NKG2A blockade with monalizumab. Together, these findings establish a therapeutic strategy that integrates transient CAR-NK engineering with immune checkpoint inhibition to overcome AML resistance, providing a safe, flexible, and clinically scalable immunotherapy platform for refractory myeloid malignancies.

## Supplementary Information

Supplementary Material 1.

Supplementary Material 2.

Supplementary Material 3.

## Data Availability

The data generated or analyzed during the current study are available within the article and supplemental information. The RNA-seq data from the FPMTB cohort were obtained from Zenodo ([https://zenodo.org/records/7274740](https://zenodo.org/records/7274740)), and data from BeatAML cohort were obtained from [https://biodev.github.io/BeatAML2/] (https://biodev.github.io/BeatAML2). The drug sensitivity dataset reported by Qin et al. was accessed as described in the original publication.

## Abbreviations

AML: Acute Myeloid Leukemia
AUC: Area Under the Curve
BLI: Bioluminescence Imaging
BMMCs: Bone Marrow Mononuclear Cells
CAR-T: Chimeric Antigen Receptor T cell
CAR-NK: Chimeric Antigen Receptor Natural Killer Cell
CB: Cord Blood
CFU: Colony-Forming Unit
CLL-1: C-type Lectin-Like Molecule-1
CR: Complete Remission
CRS: Cytokine Release Syndrome
DEGs: Differentially Expressed Genes
FDR: False Discovery Rate
GSEA: Gene Set Enrichment Analysis
HLA-E: Human Leukocyte Antigen-E
HSCs: Hematopoietic Stem Cells
ICANS: Immune Effector Cell–Associated Neurotoxicity Syndrome
IVT: In Vitro Transcription
LNP: Lipid Nanoparticle
MDR: Multidrug Resistance
mRNA: Messenger RNA
NKG2A: Natural Killer Group 2 Member A
NT-NK: Non-Treated Natural Killer Cell
PBMCs: Peripheral Blood Mononuclear Cells
sDSS: Selective Drug Sensitivity Score
UTRs: Untranslated Regions
