# Supplementary material for: Engineering CLL-1 CAR-NK cells via mRNA–LNP for potent antitumor activity and reversal of HLA-E–mediated resistance in acute myeloid leukemia
Source: J Exp Clin Cancer Res. 2026 Mar 17;45:105. doi: 10.1186/s13046-026-03689-4 (PMC13107750; doi:10.1186/s13046-026-03689-4)
Supplement: Supplementary file 1 — Supplementary Material 1. [file 13046_2026_3689_MOESM1_ESM.docx]

**Supplemental Information**

**Figure S1**


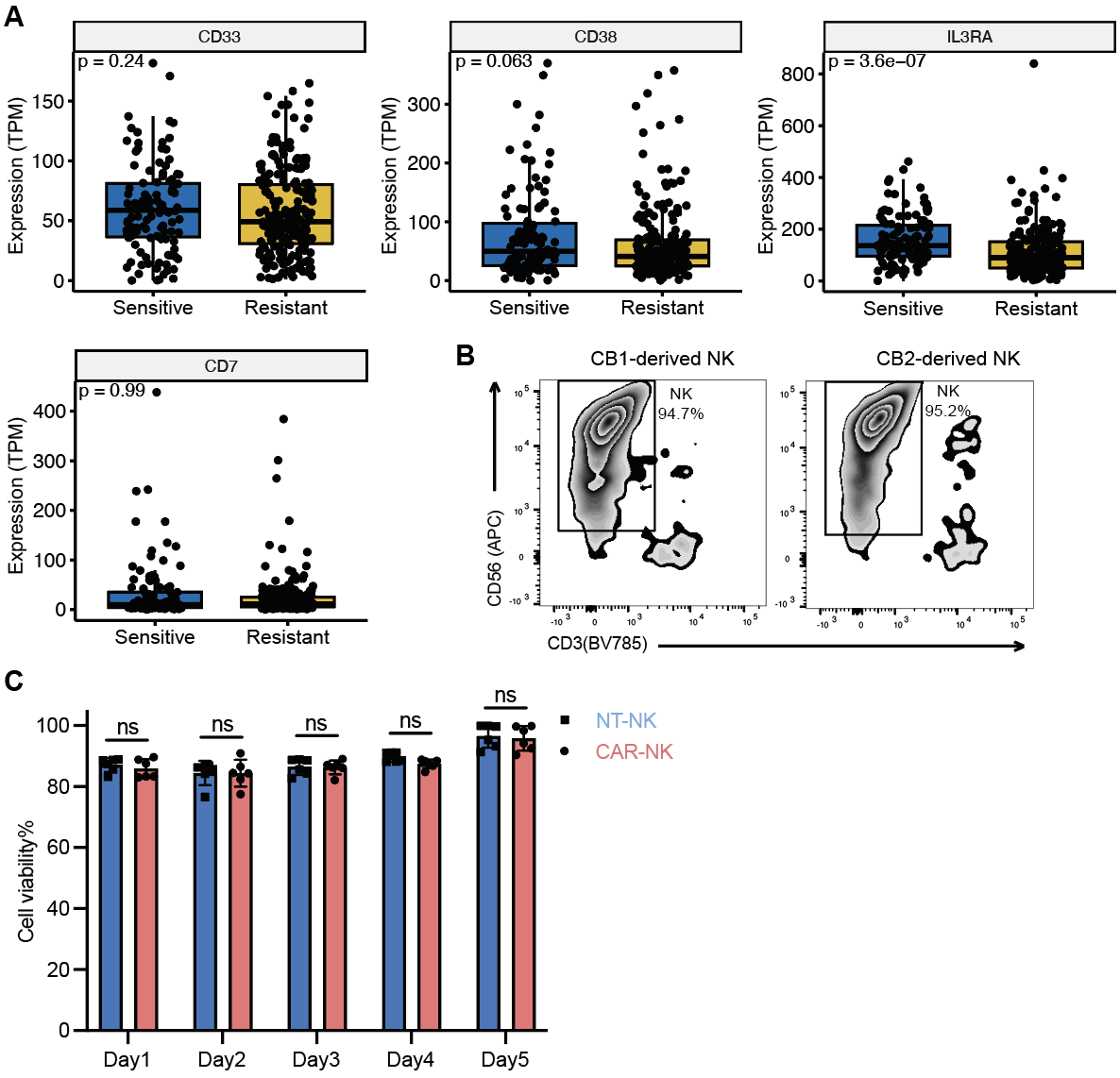


(A) Comparative analysis of CD33, CD7, IL3RA (CD123) and CD38 in MDR AML. Mann-Whitney U test. (B) Representative flow cytometry analysis of NK cell purity from two independent CB donors. NK cells were identified as CD3⁻CD56⁺ populations. (C) Viability (gated on FSC/SSC) of CLL-1 CAR-NK versus NT-NK cells during long-term culture (n = 6). Two-way ANOVA multiple comparisons. ns p > 0.05.

**Figure S2**


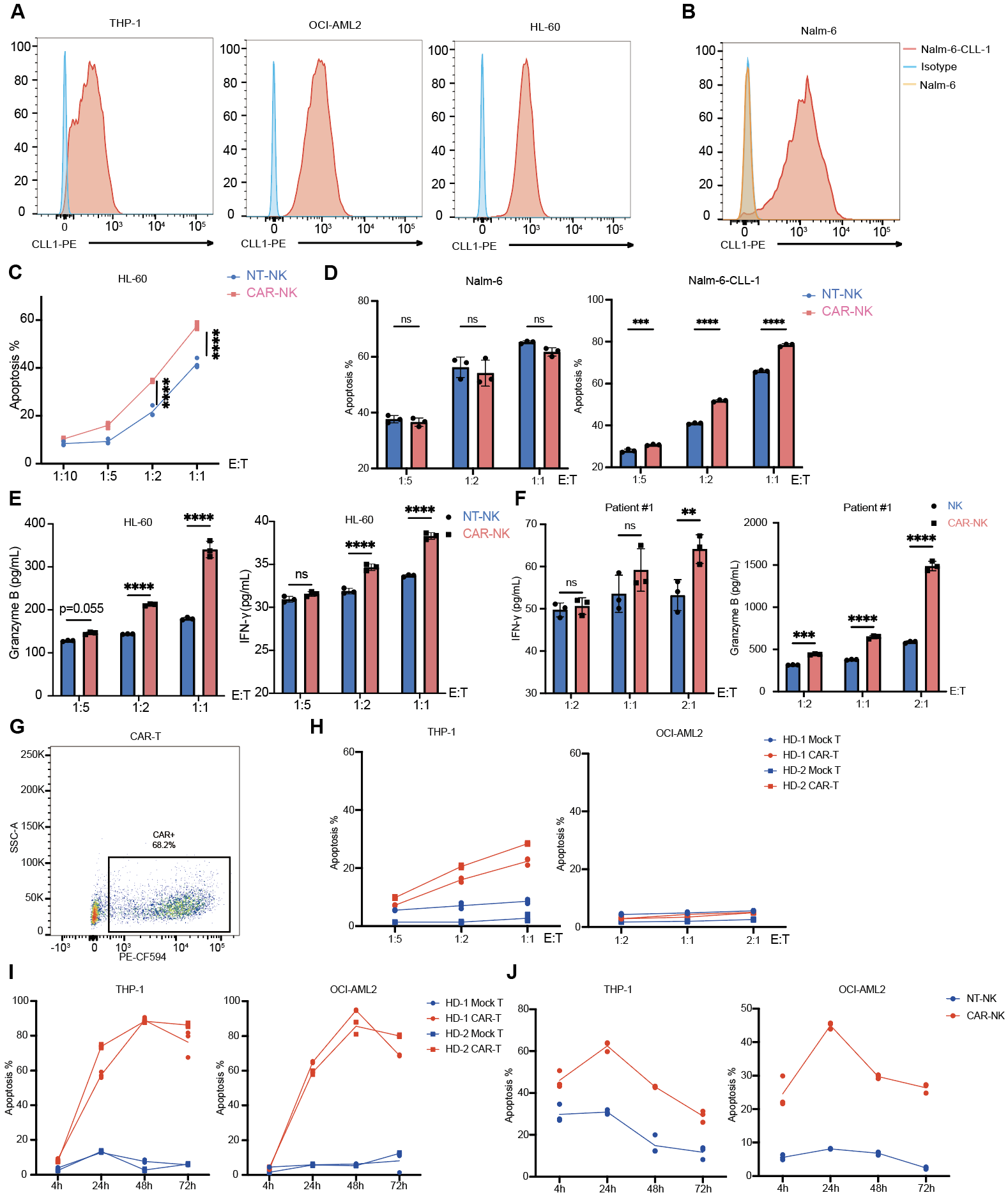


(A) Flow cytometric analysis of CLL-1 expression on the cell lines THP-1, OCI-AML2, HL-60. (B) Flow cytometric analysis of CLL-1 expression in Nalm-6 (Nalm-6-CLL-1) cells and CLL-1-overexpressing Nalm-6 cells. (C) CLL-1 CAR-NK cells lysed CLL-1^+^ cell lines HL-60. (D) Cytotoxicity of CLL-1 CAR-NK cells against Nalm-6 and Nalm-6-CLL-1 cells after 4 h co-culture at different E:T ratios. (E) CLL-1 CAR-NK cells secreted IFN-γ and Granzyme B in a dose-dependent manner against HL-60 cells, measured by ELISA (n = 3). (F) IFN-γ and Granzyme B secretion by NT-NK or CLL-1 CAR-NK cells against patient #1 target cells, measured by ELISA (n = 3). (G) Flow cytometric analysis showing CAR expression in lentiviral-transduced CAR-T cells. (H) Percentage of apoptosis in THP-1 and OCI-AML2 cells after 4 h co-culture with mock T cells or CLL-1 CAR-T cells derived from PBMCs of two healthy donors at different E:T ratios (n = 3). (I) Percentage of apoptosis in THP-1 cells at an E:T ratio of 1:5 and in OCI-AML2 cells at an E:T ratio of 1:1 after co-culture with mock T cells or CLL-1 CAR-T cells derived from PBMCs of two healthy donors for 4, 24, 48, and 72 h. (J) Percentage of apoptosis in THP-1 cells at an E:T ratio of 1:5 and in OCI-AML2 cells at an E:T ratio of 1:1 after co-culture with NT-NK or CAR-NK cells for 4, 24, 48, and 72 h. Mean ± SEM. Two-way ANOVA multiple comparisons. ****p < 0.0001, ***p < 0.001, **p < 0.01, ns p > 0.05;

**Figure S3**


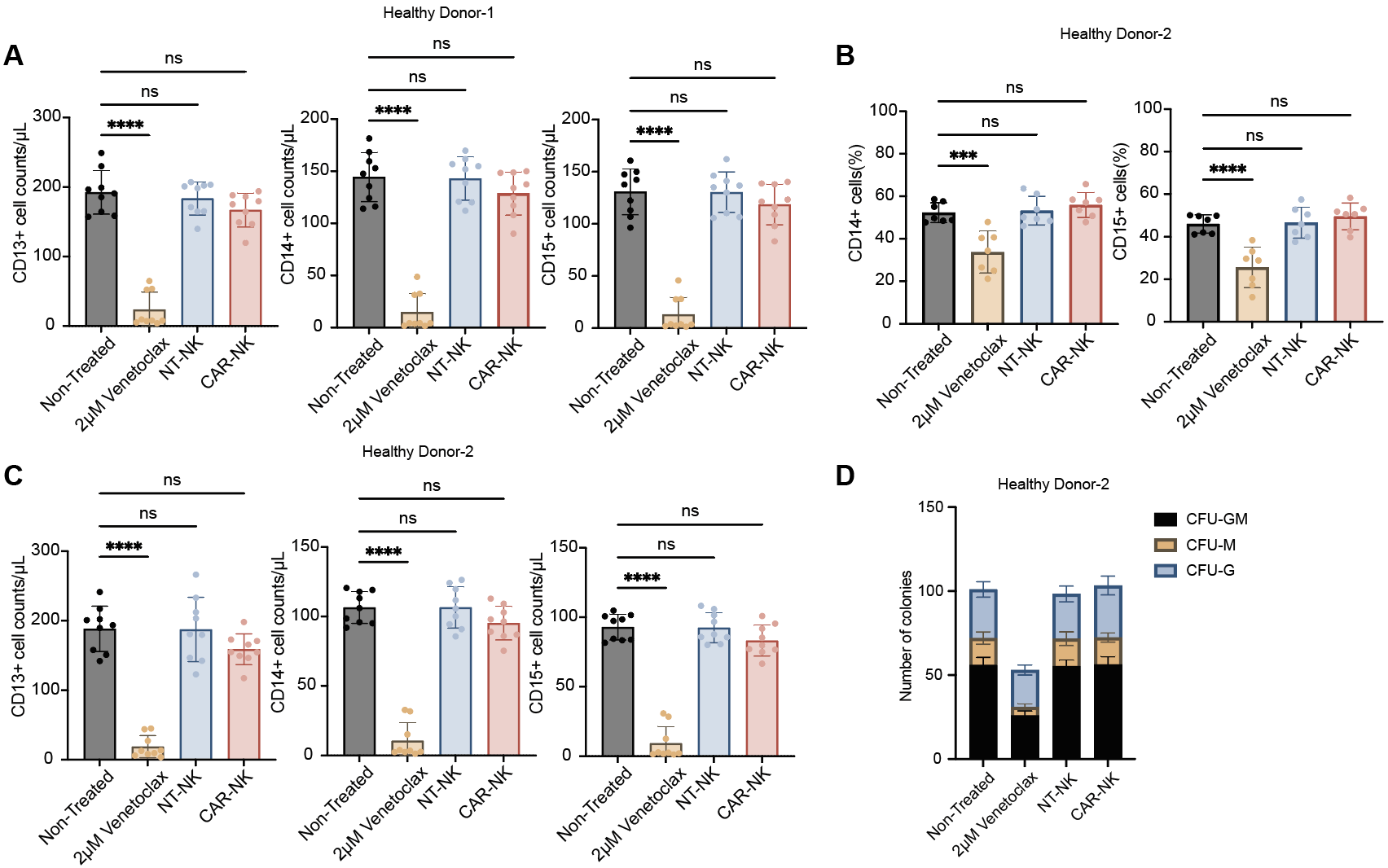


(A) Flow cytometry was performed to quantify the absolute numbers of CD13^+^, CD14^+^, and CD15^+^ cells from healthy donor-1(n = 9). (B–D) PBMCs from healthy donor-2 were co-cultured with either NT-NK cells, CLL-1 CAR-NK cells, or medium alone at an E:T ratio of 1:1 for 24 h, or with 2 μM venetoclax for 24 h. CD34^+^ cells were then sorted by FACS and plated in HemaTox myeloid medium for 7 days or in semisolid methylcellulose-based growth medium for 14 days, followed by colony counts for CFU-G, CFU-M, and CFU-GM colonies (D) (n = 9). Flow cytometry was performed to quantify the percentage (B) and the absolute counts of CD13^+^, CD14^+^, and CD15^+^ cells (C) (n = 9). one-way ANOVA multiple comparisons. Mean ± SD. ****p < 0.0001, ***p < 0.001, ns p > 0.05;

**Figure S4**


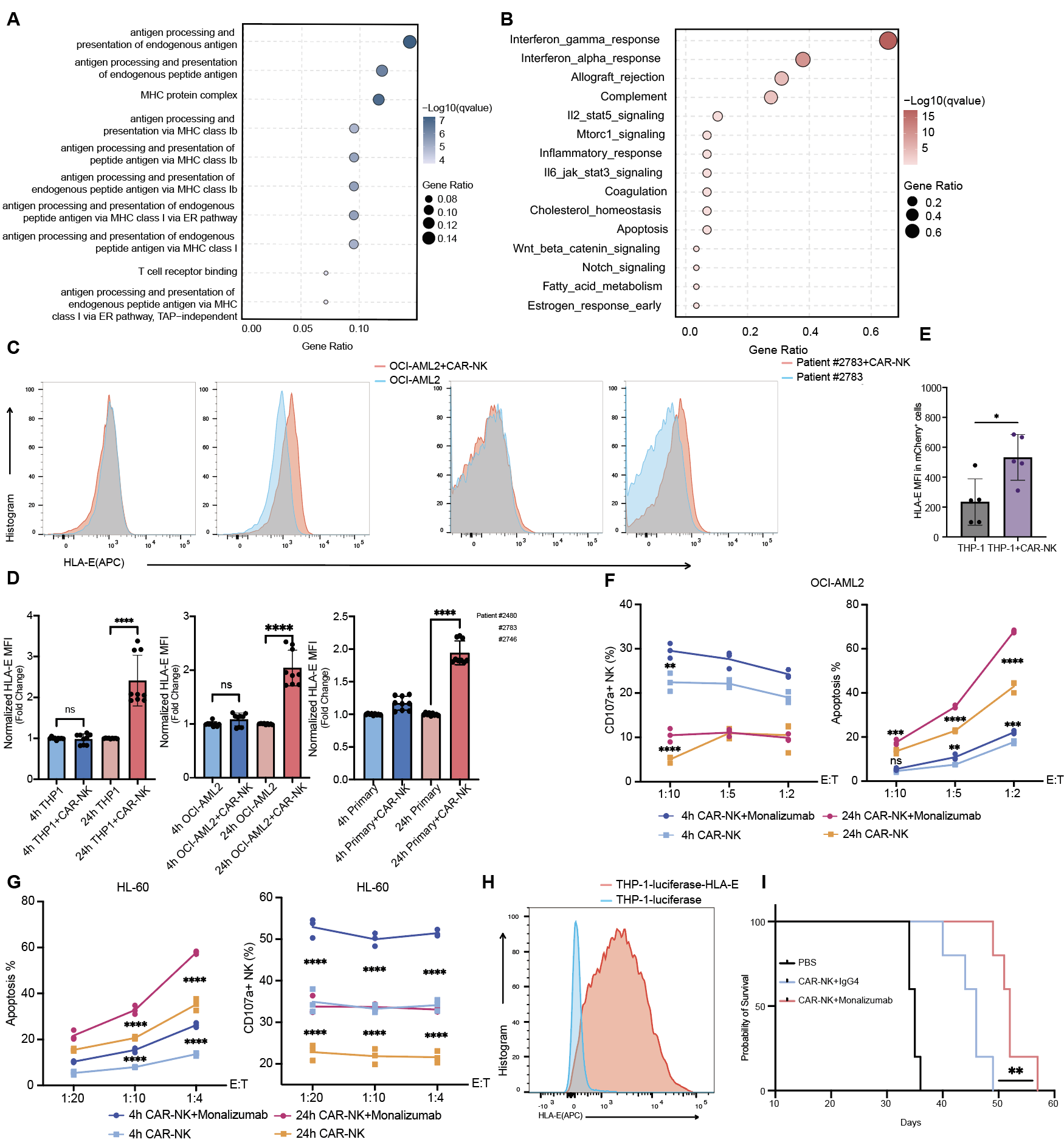


(A, B) Gene ontology (A) and HALLMARK (B) pathway enrichment analysis of the 46 overlapping upregulated genes identified in Figure 5B. (C) Representative flow cytometry histograms showing HLA-E surface expression on OCI-AML2 (E:T = 1:1) and primary cells from patient #2783 (E:T = 1:2) cultured alone or with CAR-NK cells for 4 or 24 h. (D) HLA-E surface expression on THP-1 (E:T = 1:5), OCI-AML2 (E:T = 1:1), and primary cells from three AML patients (E:T = 1:2) cultured alone or co-cultured with different CB-derived CAR-NK cells for 4 or 24 h (n = 9). (E) HLA-E surface expression on mCherry^+^ tumor cells in bone marrow (n = 5). (F–G) Flow cytometric analysis of percentage of CD107a^+^ CAR-NK cells and the apoptosis of OCI-AML2 and HL-60 cells after 4 or 24 h co-culture with CAR-NK cells in combination with isotype control IgG4 or the anti-NKG2A monoclonal antibody Monalizumab (5 μg/mL) (n = 3). (H) Representative flow cytometry histograms showing HLA-E surface expression on THP-1-Luc^+^ and THP-1-Luc^+^-HLA-E cells. (I) Kaplan–Meier curves of mice treated with PBS, CAR-NK combined with IgG4 control, or CAR-NK combined with monalizumab (n = 5). Log-rank test. Two-way ANOVA for multiple comparisons. Mean ± SD; ****p < 0.0001, ***p < 0.001, **p < 0.01, *p < 0.05, ns p > 0.05.

**Figure S5**


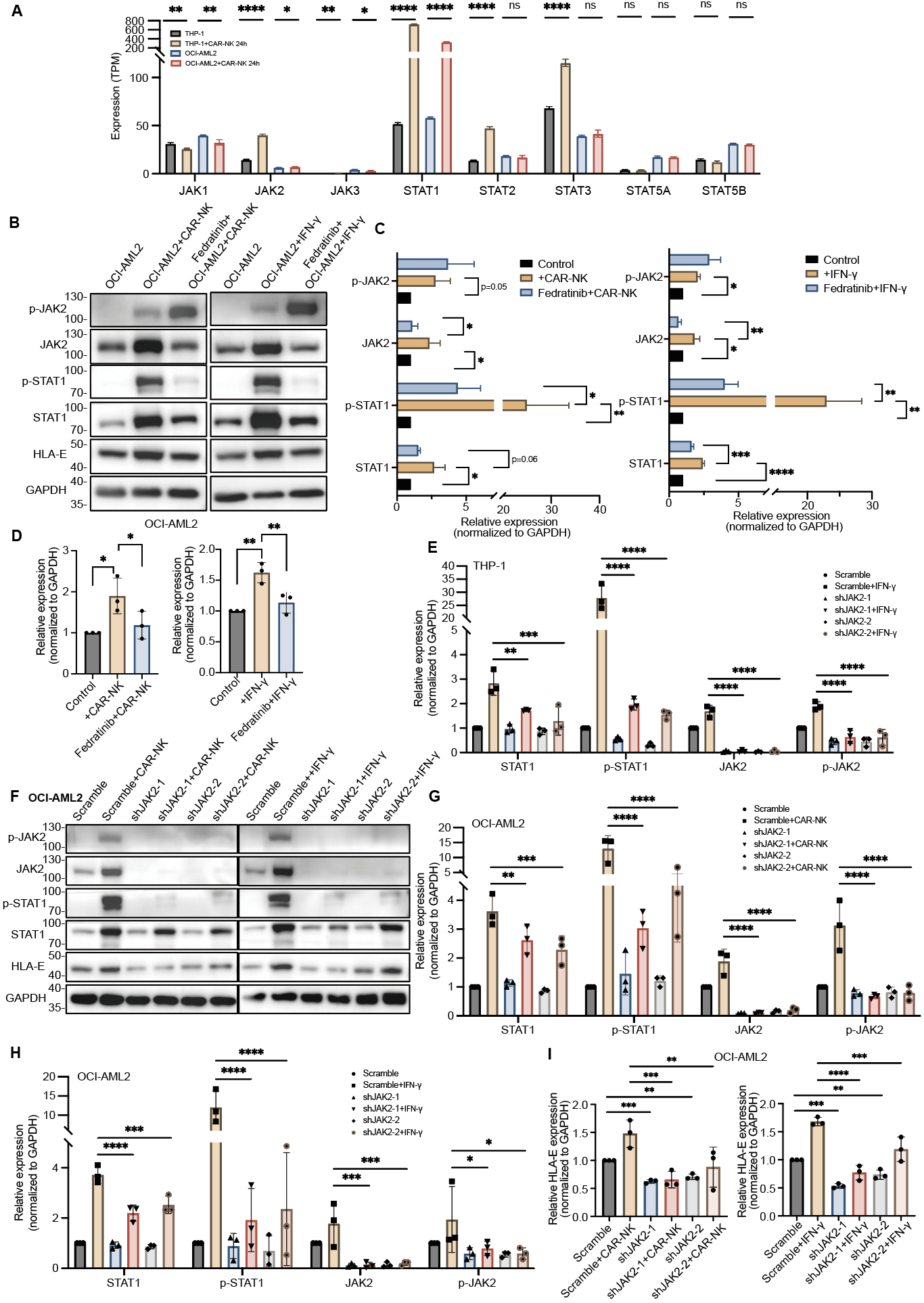


(A) TPM expression levels of the JAK–STAT family genes from RNA-seq analysis (Figure 5A). (B) Representative Western blots of JAK2–STAT1 pathway activation and HLA-E expression in OCI-AML2 cells (E:T = 1:1) co-cultured with CAR-NK cells or treated with IFN-γ (800 U/mL) for 24 h in the presence or absence of Fedratinib (500 nM). (C, D) Densitometric quantification normalized to GAPDH of JAK2–STAT1 signaling (C) and HLA-E protein expression (D) from three independent experiments in OCI-AML2 (n = 3). (E) Densitometric quantification normalized to GAPDH of JAK2–STAT1 signaling from three independent experiments in THP-1 treated with IFN-γ (800 U/mL) (n = 3). (F) Representative Western blots of JAK2–STAT1 pathway activation and total HLA-E expression in OCI-AML2 cells expressing scramble control or JAK2 shRNA after 24 h CAR-NK co-culture (E:T = 1:1) or IFN-γ treatment (800 U/mL). (G–I) Densitometric quantification normalized to GAPDH of JAK2–STAT1 signaling (G, H) and HLA-E expression (I) from three independent experiments in OCI-AML2 (n = 3). statistical significance determined by two-way ANOVA for multiple comparisons. Mean ± SD. ****p < 0.0001, ***p < 0.001, **p < 0.01, *p < 0.05, ns p > 0.05;


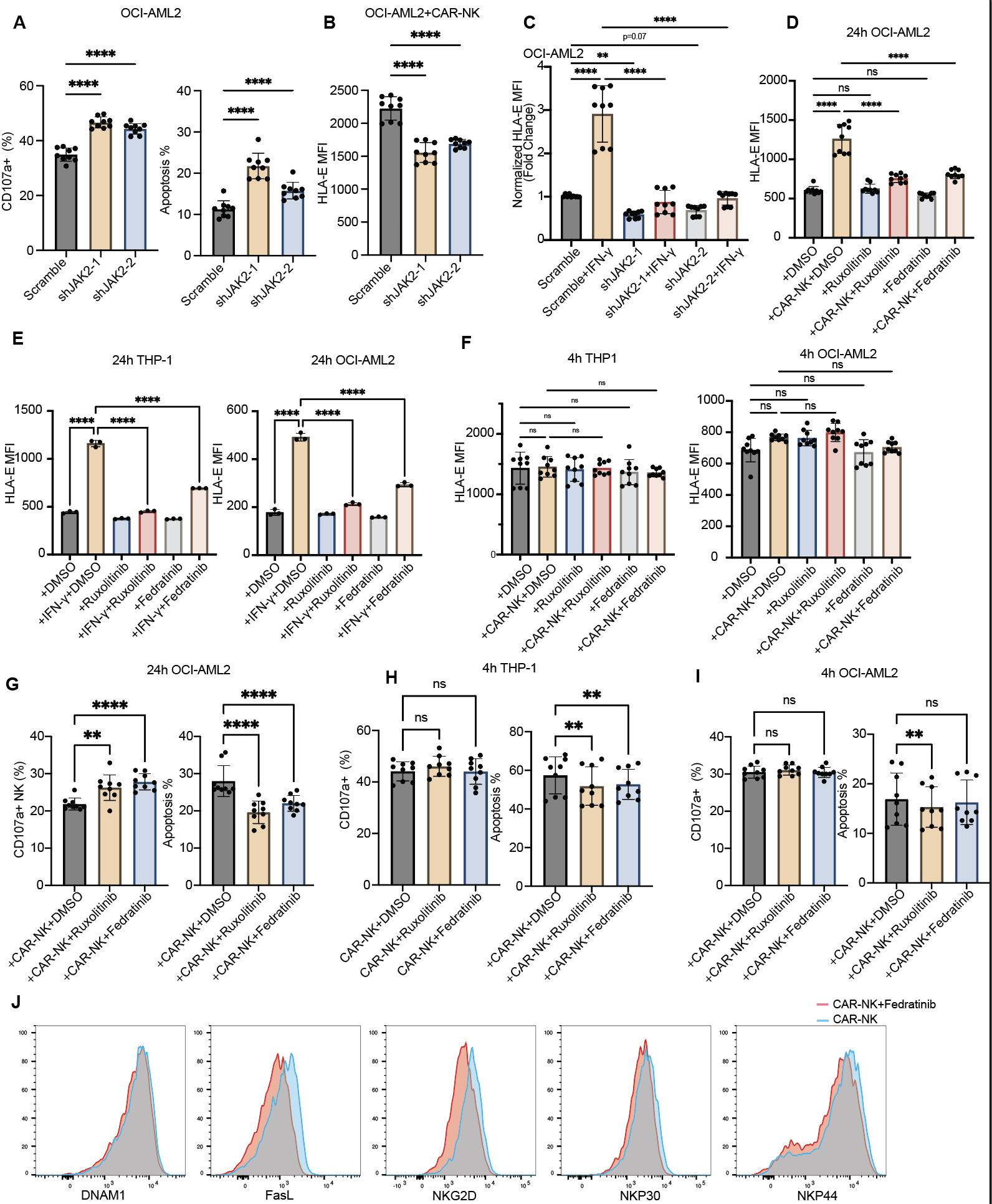


(A) Flow cytometric analysis of CAR-NK CD107a degranulation and OCI-AML2 apoptosis after 4 h co-culture with scramble or JAK2-knockdown OCI-AML2 cells (n = 9). E:T = 1:1. (B, C) Flow cytometry of surface HLA-E expression in scramble vs. JAK2-knockdown OCI-AML2 cells following 24 h co-culture with CAR-NK cells (E:T = 1:1, B) or IFN-γ treatment (800 U/mL, C) (n = 9). (D) Comparison of HLA-E surface MFI in OCI-AML2 cells after 24 h CAR-NK co-culture (E:T = 1:1) with or without Ruxolitinib or Fedratinib (500 nM) (n = 9). (E) Comparison of HLA-E surface MFI in THP-1 and OCI-AML2 cells after 24 h IFN-γ treatment (800 U/mL) with or without Ruxolitinib or Fedratinib (500 nM) (n = 3). (F) Comparison of HLA-E surface MFI in THP-1 (E:T = 1:5) and OCI-AML2 (E:T = 1:1) cells after 4 h CAR-NK co-culture with or without Ruxolitinib or Fedratinib (500 nM) (n = 9). (G) CAR-NK CD107a degranulation and OCI-AML2 apoptosis after 24 h co-culture in the presence or absence of Ruxolitinib or Fedratinib (500 nM) (n = 9). E:T = 1:1. (H, I) CAR-NK CD107a degranulation and THP-1 (E:T = 1:5) and OCI-AML2 (E:T = 1:1) apoptosis after 4 h co-culture in the presence or absence of Ruxolitinib or Fedratinib (500 nM) (n = 9). (J) Representative flow cytometry histograms showing expression of activating receptors on CAR-NK cells after 24 h culture with Fedratinib (500 nM) (n = 3). two-way ANOVA for multiple comparisons. Data represent mean ± SD; statistical significance determined by two-way ANOVA for multiple comparisons. ****p < 0.0001, ***p < 0.001, **p < 0.01, ns p > 0.05.

**Methods and materials**

| Reagent / Resource | Source / Company | Identifier / Catalog Number |
| --- | --- | --- |
| hIL-2 | PeproTech | 100 IU/mL |
| Anti-CD56-APC / PE/Cy7 | Biolegend | 318309 / 318317 |
| Anti-CD3-FITC / BV785 | Biolegend | 300406 / 317330 |
| Anti-CD16-FITC | Biolegend | 302006 |
| Anti-CD57-PE/Cy7 | Biolegend | 359624 |
| Anti-NKG2C-PE | Biolegend | 375004 |
| Anti-NKp30-PE | Biolegend | 325207 |
| Anti-NKp44-PE | Biolegend | 325107 |
| Anti-NKp46-PE/Cy7 | Biolegend | 331916 |
| Anti-NKG2A-PE | Biolegend | 375104 |
| Anti-NKG2D-PE/Cy7 | Biolegend | 320812 |
| Anti-HLA-E-APC | Biolegend | 342606 |
| Anti-CD107a-PE/Dazzle 594 / PE | Biolegend | 328646 / 328608 |
| Anti-CLL-1-PE / APC | Biolegend | 353604 / 353606 |
| Anti-CD13-APC | Biolegend | 301706 |
| Anti-CD14-FITC | Biolegend | 325604 |
| Anti-CD15-PE | Biolegend | 301905 |
| Anti-FasL-PE | Biolegend | 306407 |
| Anti-DNAM-1-PE | Biolegend | 338305 |
| Anti-TIGIT-PE/Cyanine7 | Biolegend | 372713 |
| Anti-LAG3-PE | Biolegend | 369305 |
| Annexin V – FITC / APC / APC/Fire 750 | Biolegend | 640906 / 640920/ 640953 |
| Precision counting beads | Biolegend | 424902 |
| Alexa Fluor 647-conjugated AffiniPure F(ab’)_2_ Fragment Goat Anti-Mouse IgG | Jackson Immunoresearch | 115-606-072 |
| Western blot antibodies | Cell Signaling Technology / ABclonal | JAK2: 3230S  p-JAK2: 3776S  STAT1: 14994S  p-STAT1: 7649S  HLA-E: A8429  GAPDH: 8884S |
| JAK2 shRNA target |  | shJAK2-1:  GCTTTGTCTTTCGTGTCATTA  shJAK2-2:  GCAGAATTAGCAAACCTTATA |
| IFN-γ / Granzyme B ELISA kits | Dayou / Dakewe | 1110002 / 1118502 |
| Monalizumab / IgG4 | MCE | HY-P99032 / HY-P99003 |
| Ruxolitinib / Fedratinib | Selleck | S1378 / S2736 |
